# Allelome.PRO, a pipeline to define allele-specific genomic features from high-throughput sequencing data

**DOI:** 10.1093/nar/gkv727

**Published:** 2015-07-21

**Authors:** Daniel Andergassen, Christoph P. Dotter, Tomasz M. Kulinski, Philipp M. Guenzl, Philipp C. Bammer, Denise P. Barlow, Florian M. Pauler, Quanah J. Hudson

**Affiliations:** CeMM Research Center for Molecular Medicine of the Austrian Academy of Sciences, Lazarettgasse 14, AKH BT 25.3,1090 Vienna, Austria

## Abstract

Detecting allelic biases from high-throughput sequencing data requires an approach that maximises sensitivity while minimizing false positives. Here, we present Allelome.PRO, an automated user-friendly bioinformatics pipeline, which uses high-throughput sequencing data from reciprocal crosses of two genetically distinct mouse strains to detect allele-specific expression and chromatin modifications. Allelome.PRO extends approaches used in previous studies that exclusively analyzed imprinted expression to give a complete picture of the ‘allelome’ by automatically categorising the allelic expression of all genes in a given cell type into imprinted, strain-biased, biallelic or non-informative. Allelome.PRO offers increased sensitivity to analyze lowly expressed transcripts, together with a robust false discovery rate empirically calculated from variation in the sequencing data. We used RNA-seq data from mouse embryonic fibroblasts from F1 reciprocal crosses to determine a biologically relevant allelic ratio cutoff, and define for the first time an entire allelome. Furthermore, we show that Allelome.PRO detects differential enrichment of H3K4me3 over promoters from ChIP-seq data validating the RNA-seq results. This approach can be easily extended to analyze histone marks of active enhancers, or transcription factor binding sites and therefore provides a powerful tool to identify candidate *cis* regulatory elements genome wide.

## INTRODUCTION

Mammalian cells are diploid and thus contain two copies of every gene locus, one inherited from the male, and one from the female parent. Mitochondrial genes, plus genes on the sex chromosomes in males, are the only exception to this rule. Since each diploid gene locus has the possibility to be expressed independently from either parental chromosome, different allelic states of expression can arise. The majority of mouse genes are considered to show equal or ‘biallelic’ expression from both parental alleles based on the absence of parental-specific phenotypes in the majority of genes analyzed by gene knockout (1). Genes that deviate from biallelic expression by showing preferential expression of one of the two parental alleles are described as showing ‘monoallelic’ expression. To date, only a small subset of mammalian genes is known to show monoallelic expression. When either parental allele can show preferential expression, this is known as random monoallelic expression (RMAE). However, when one parental allele consistently and heritably shows preferential expression, this is known as parental-specific or imprinted monoallelic expression (IMAE).

Random monoallelic expression has been shown to affect clustered gene families, such as the allelic exclusion of the B- and T-cell receptor genes that allows clonal lymphocytes to express a single receptor with a unique specificity (2), the ‘singular’ expression of the clustered olfactory receptor genes that allows neurons to discriminate olfactory signals (3), and more recently, the stochastic monoallelic expression of the cadherin-related PCDH neuronal receptor clusters that may act in neuronal self recognition (4). All X-chromosome linked genes in female placental mammals also show random monoallelic expression, due to RMAE of a single locus containing the *Xist* long non-coding (lnc) RNA, which controls X-chromosome inactivation (5). The X-chromosome can also display imprinted paternal-specific inactivation in some rodent extra-embryonic tissues, due to preferential paternal expression of the *Xist* lncRNA (6). In all these cases, RMAE can occur in inbred mouse strains, and thus can be initiated from genetically identical parental alleles, indicating an epigenetic mechanism.

In contrast to the clustered gene families mentioned above that use RMAE to generate specificity in clonal cells, up to 10% of solo autosomal genes were reported to show RMAE in isolated cell lines that could be stably propagated (7,8). Similarly, an estimated 12–24% of expressed genes showed monoallelic gene expression in single cells of F1 mouse pre-implantation embryos, indicating that this could be a widespread phenomenon that may play a role in generating diversity in individual cells (9). In cases of true RMAE an important point to bear in mind is that, although a gene may show monoallelic expression that can be detected by single cell assays, at the population level the gene will appear biallelic if the allele expressed is random in each cell.

In addition to IMAE and RMAE, a third category of monoallelic expression that may occur in outbred individuals is non-random monoallelic expression or strain bias. Such strain bias may occur due to genetic differences between the alleles that affect expression of certain genes. For example, expression differences could arise from nucleotide polymorphisms influencing the interaction of promoters and enhancers with transcription factors and thereby affecting transcription rates. Such polymorphisms could also act at a post-transcriptional level by influencing miRNA binding and RNA stability, or allele-specific processing, such as alternative splicing or alternative UTR generation (10–12). The *Xist* lncRNA that controls X-chromosome inactivation in female cells can also show a strain bias due to genetic variation at the X-inactivation center (*Xic*) locus that influences the likelihood of *Xist* being expressed from that chromosome (13). *Mus musculus castaneus* (CAST/EiJ) mice are known to possess a stronger *Xic* allele than *Mus musculus domesticus*, thus in the FVB/N x CAST/EiJ reciprocal crosses used in our study, the FVB/N X-chromosome will be preferentially inactivated (13,14).

Imprinted monoallelic expression primarily affects small clusters of unrelated genes (15). Currently 96 of the 123 known imprinted genes either lie in genetically characterized imprinted clusters, or, due to their close proximity are likely to lie in clusters (Supplementary Table S2). Thus most imprinted genes are clustered. A novel feature of several gene clusters showing IMAE in contrast to those showing RMAE, is their association with a long non-coding (lnc) RNA (16–18), that in four cases has been shown to induce imprinted gene silencing (reviewed in (15)). While some solo genes clearly show imprinted expression, the imprinted status of many has been challenged (19–21). Thus, the number of solo imprinted genes is not yet known. The defining characteristic of an imprinted gene is preferential expression from one parental chromosome. However, the exact ratio of parental-specific expression that constitutes imprinted expression has not yet been defined. The total number of known imprinted genes is also relatively low, only ∼0.5% of protein-coding genes and approximately equal numbers of maternally-expressed and paternally-expressed imprinted genes are known. This total number of imprinted genes was obtained from examination of a limited set of tissues such as embryo, placenta and fetal brain that are predicted to use imprinted gene expression to regulate pre- and post-natal growth of the mammalian embryo (22–24). However, it has only recently been appreciated that imprinted expression shows considerable tissue-specificity (25) and also developmental regulation (15). Given that only a limited number of tissues and developmental stages have been assayed so far, and even fewer studies of different mammalian taxonomic strains conducted, it is not known if the total number of imprinted genes has been underestimated. This possible underestimation of the total number of imprinted genes has implications for understanding the biological function of imprinted gene expression in mammals.

In recent years many studies have used high-throughput RNA sequencing (RNA-seq) of tissues from reciprocal crosses between genetically distinct inbred mouse strains to identify imprinted expression (26–30). These studies based on a few tissue types only found a small number of novel imprinted genes compared to those listed in publically available databases (www.otago.ac.nz/IGC). In contrast, one study reported parental-specific expression of 1300 transcripts in embryonic and adult mouse brain (31). However, a subsequent study indicated that the vast majority of these transcripts were false positives, and emphasized the need for careful controls including the use of biological replicates, the need to empirically determine the false positive rate, and the need for independent validation of the imprinted status of the gene (32). With these three requirements in mind, we developed Allelome Profiler (Allelome.PRO), an automated and user-friendly bioinformatic pipeline based on a previously described method (32,33), but modified to improve the robustness and sensitivity of imprinted expression detection, and also to detect strain bias gene expression as well as biallelically expressed and silent genes. Critically, in addition to a false discovery rate cutoff based on a statistical score, we introduced an allelic ratio cut-off for both parental and strain bias that removes loci showing a minor allelic bias with high sequencing coverage, thus enabling the allelic status of all genes to be categorised. This cut-off was determined from the expression patterns of known imprinted genes and from X-linked genes on X-chromosomes showing skewed X-inactivation. We use primary mouse embryo fibroblasts (MEFs) and we define different allelic states of expression as imprinted, strain-biased, biallelic, non-informative (due to low or no expression) or having no single nucleotide polymorphisms (SNPs). We also show that Allelome.PRO can detect allelic differences in high-throughput chromatin immunoprecipitation sequencing (ChIP-seq) data and demonstrate that H3K4me3, a promoter mark associated with active transcription, can be used as an independent validation of the RNA-seq allelome. Together this approach allows a high-resolution analysis of the entire allelome of any cell type and has the potential to expand our understanding of genetic and epigenetic mechanisms underlying IMAE, RMAE and the phenotypic differences between strains.

## MATERIALS AND METHODS

### Generation of mouse embryonic fibroblasts (MEFs)

CAST/EiJ (CAST) mice were purchased from the Jackson Laboratory (www.jax.org) and FVB/NJ (FVB) from Charles River to generate reciprocal crosses. After reciprocal mating (CASTxFVB and FVBxCAST), mouse embryonic fibroblasts (MEFs) were derived from E12.5 embryos after removing the head, viscera and urogenital system. The remaining carcass was homogenised to a single cell suspension using Trypsin/EDTA (Gibco) and plated on 6 cm dishes. Female MEFs from passage 2 of a confluent 10 cm plate were used for the RNA analysis, whereas MEFs from passage 5 of three confluent T175 cm^2^ flasks were used for ChIP. The sex of the embryos was determined by PCR combining a Y-chromosome specific assay and an autosomal assay (34).

### RNA and ChIP-seq sample preparation

Total RNA and DNA were extracted using TRI-reagent (Sigma–Aldrich T9424) according to the manufacturers protocol. Total RNA was DNaseI treated using the DNA-Free™ kit (Ambion). Ribosomal RNA was depleted from total DNaseI-treated RNA using the RiboZero rRNA removal kit (Human/Mouse/Rat) (Epicentre). Strand-specific RNA-seq libraries were prepared employing the TruSeq RNA Sample Prep Kit v2 (Illumina) modified as described for strand-specific sequencing (35). Native ChIP for H3K4me3 (antibody: cat. 07-473, lot 2019729, Millipore) was conducted as described (36). ChIP-seq libraries were prepared using the TruSeq ChIP Sample Prep Kit (Illumina). 100 bp paired end sequencing for RNA-seq and 50 bp single end sequencing for ChIP-seq were performed by the Biomedical Sequencing Facility (BSF) in Vienna using the Illumina HiSeq 2000 platform.

### Alignment of sequencing data

Raw RNA sequencing data was aligned using STAR (version 2.3.1z12) (37), GSNAP (version 2014.07.04) (38) and TopHat (version 2.0.12, bowtie 2.2.3) (39) to allow a comparison between the three aligners. Reads mapping to multiple locations were excluded using specific parameters (STAR: –outFilterMultimapNmax 1), combining only output files that contained uniquely aligned reads (GSNAP), or by removing secondary alignments identified by the SAM flag (TopHat). Additional parameters for the STAR alignment were a maximum intron size of 100 000 bp and out-filtering of non-canonical splice junctions. SNP-tolerant alignment was enabled for GSNAP by providing information about the SNP variants between the two crosses. TopHat was run using a RefSeq based transcriptome index and parameters chosen to exclude novel junctions as well as novel insertions and deletions. As sequencing was done in a strand specific manner, the aligned reads were subsequently separated according to strand using a custom Perl script. STAR alignment of ChIPseq data was conducted with different parameters to disable spliced reads, i.e. a maximum intron size of 1, and prevent soft clipping by enforcing end-to-end alignment (–alignEndsType EndToEnd). All aligned BAM files were sorted afterwards using SAMtools (version 0.1.19).

### Preparation of annotation files

The NCBI RNA reference sequences collection (RefSeq) annotation was downloaded from the UCSC genome browser on 2 July 2014. Transcripts <100 bp were removed and the remaining transcripts were separated by transcriptional orientation and used for strand specific analysis of RNA-seq by Allelome.PRO. An annotation of ±2 kb windows around the transcription start site (TSS) of RefSeq annotations was used to analyze ChIP-seq by Allelome.PRO. Sliding window annotations for the whole genome were created using makewindows from the BEDtools suite (version 2.20.1).

The Single Nucleotide Polymorphism (SNP) annotation file was created from a VCF file containing SNP variant data of 18 mouse strains, downloaded from the Sanger institute (40). SNP information for the strains CAST/EiJ and FVB/NJ were extracted and converted to the required browser extensible data (BED) format using a custom script that is available together with Allelome.PRO (details see manual, Supplementary Material). Note that the name field in this file contains SNP information. Only homozygous high quality SNPs were used and SNPs overlapping annotated pseudogenes were removed.

### Reference list of imprinted genes

We defined the list of known imprinted genes by first merging the lists provided by the Harwell and Otago databases (http://www.mousebook.org/imprinting-gene-list, http://igc.otago.ac.nz, (41–43)). Genes that were not annotated by the RefSeq or UCSC database were then removed. We defined multiple imprinted isoforms from the same gene, and groups of closely linked lncRNAs with the same reported imprinted expression pattern to be a single imprinted gene. These cases are indicated in Supplementary Table S2 where we define 123 known imprinted genes. The Allelome.PRO results for RNA-seq and ChIP-seq for these genes are also included in this table, together with information from the literature including where imprinted expression is reported to occur, and if the imprinted status of the gene is disputed.

### Saturation curves

Saturation curves were created by Allelome.PRO runs on random sampled subsets of aligned reads. Random sampling was performed using the Picard toolset (version 1.111) for sampling rates of 5, 10, 15, 20, 25, 30, 35, 40, 50, 60, 70, 80 and 90% of total reads. Three technical replicates were produced using different random seeds (1, 2 and 3). Reads were separated according to the transcribed strand after sampling to allow strand-specific analysis by Allelome.PRO. Basic statistical analysis of the resulting data was performed using R (44).

### Simulation of sequencing errors

Aligned RNA-seq reads from the region surrounding the *Igf2r* imprinted cluster (GRCm38/mm10 chr17:12350000–13000000) were extracted from the BAM files of the two forward and two reverse FVB/CAST MEF crosses and converted to FASTQ format. With a custom made Perl script we randomly generated errors for each base in a read at different frequencies (1%, 5%, 10% or 15%), and repeated this three times. Then we re-aligned the FASTQ files and ran Allelome.PRO.

### Determining experimental error from *in silico* mixing of CAST/EiJ and FVB/N reads

100bp paired end RNA-seq data from FVB/N adult heart and CAST/EiJ adult heart was aligned to the reference genome using STAR. For CAST 85.3% reads were uniquely aligned, while for FVB 86.7% reads were uniquely aligned. To create *in silico* the two forward and two reverse crosses needed to input into Allelome.PRO, we took aliquots of reads from each strain and then combined them. Based on the alignment rate we calculated the number of input reads needed to uniquely align 3 million reads for each strain, and took sequentially four aliquots of this amount of reads from the FASTQ file (the FASTQ file lists the reads as they come off the Illumina machine, and therefore the order should be random), and then combined FVB and CAST aliquots to create the four technical replicates. We aligned the four technical replicates using STAR, assigned two replicates as forward and two as reverse crosses, and then used Allelome.PRO to calculate allelic expression. By combining equal numbers of CAST and FVB reads we expect most genes to have an allelic ratio around 0.5, but strain bias genes will show unequal ratios. However, we do not expect to find any imprinted genes. Therefore, we defined a false discovery rate (FDR) for imprinted expression as the percentage of informative genes (biallelic, strain bias, imprinted) called imprinted. We determined the FDR with no allelic ratio cutoff (plotted as 0.5) and at allelic ratio cutoffs of 0.6, 0.7, 0.8, 0.9 and 1.0, and at minread settings of 1, 2 and 3 (the minread parameter of Allelome.PRO defines the minimum number of reads over a SNP required before it is included in the analysis).

## RESULTS

### Allelome.PRO requirements

**Figure 1.**
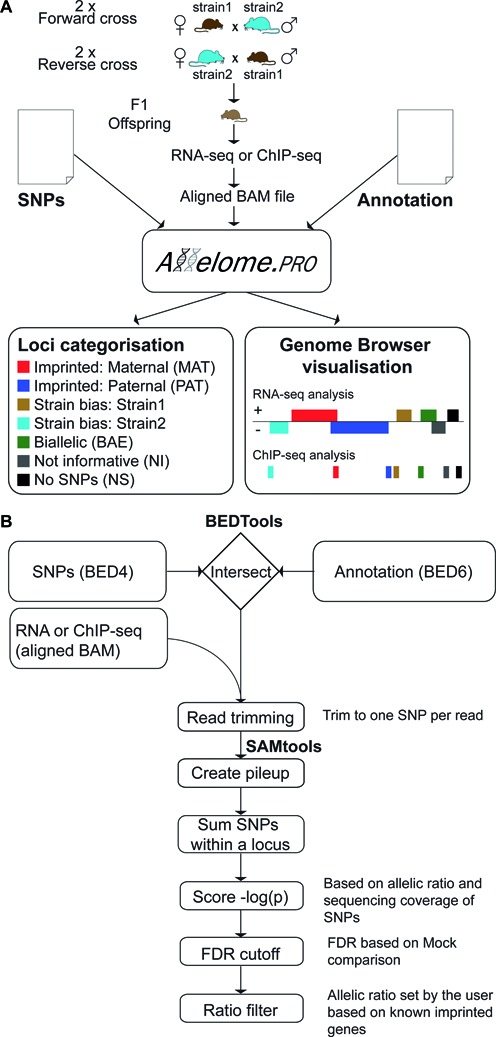
Allelome.PRO workflow to detect allele-specific genome features using RNA-seq and ChIP-seq data. (**A**) Allelome.PRO requires three types of input files: A SNP file (BED6), an annotation file (BED6) and 4 aligned BAM files from F1 reciprocal crosses (2 each of forward and reverse cross). The output categorizes the candidates in the annotation file into the following seven categories: Imprinted: MAT, maternally expressed (red) and PAT, paternally expressed (blue); Strain-biased: Strain1 expressed (brown) and Strain2 expressed (turquoise); BAE, biallelic expression (green); NI, non-informative (e.g. due to low coverage) (gray); NS, no SNP located inside the locus (black). Allelome.PRO further provides a result file (BED6) that can be uploaded to the UCSC genome browser for visual inspection. (**B**) The Allelome.PRO algorithm starts by using BEDtools to intersect the SNP file with the annotation file. The resulting intersection of SNPs located within the annotated candidates is then used to filter out aligned reads that do not overlap any of these SNPs. In the next step, a 1:1 relationship between aligned reads and SNPs is established by trimming the reads so that each read overlaps just one SNP. Subsequently a pileup file of reads at the SNP positions is created using SAMtools. Read counts for the two alleles are summed up over all SNPs within a locus. A binomial distribution is used to assess the significance of the observed allelic biases, and the resulting allelic score is defined as the negative logarithm of the derived *P* value (–log_10_(*P*)). An allelic score cutoff based on a user-set false discovery rate (FDR) is then empirically calculated using mock comparisons. The final allelic ratio cutoff filters out remaining candidates with an allelic ratio below a user-set limit (see the manual for details, Supplementary material).

The Allelome Profiler (Allelome.PRO) pipeline uses custom Perl, shell and R scripts to analyze allelic specific features in massive parallel DNA sequencing data (see manual, Supplementary Material). Allelome.PRO is designed for Linux based operating systems and uses efficient software suites to optimize both the runtime and memory footprint, with SAMtools and BEDtools being the only dependencies (45,46). The Allelome.PRO pipeline depends on data obtained from genetically distinct individuals or pooled samples from two strains and requires three files to be provided by the user in order to start the fully automated analysis (Figure 1A). First, a file defining single nucleotide polymorphisms (SNPs) between the two strains is required in browser extensible data (BED4) format (note the special requirements for the name field detailed in the methods). Second, an annotation file defining the genomic regions to be analyzed must be provided in BED6 format (i.e. a BED file with six fields as defined on http://genome.ucsc.edu, (47)). Overlapping regions with identical names (fourth field in the BED file) are merged into single loci. Finally, aligned sequencing data must be provided as an aligned compressed binary version of the Sequence Alignment Map (BAM) file (45). Allelome.PRO is capable of analysing any DNA sequencing data, however we have tested and optimized it for the massive parallel sequencing of cDNA ends (RNA-Seq) and of chromatin immunoprecipitation (ChIP-Seq). In order to apply statistical testing for allele specific enrichment Allelome.PRO requires four biological samples for RNA-Seq or ChIP-Seq. These are two replicate samples from the F1 offspring of a forward cross between strain1 (mother) and strain2 (father), and two replicates from a reverse cross, where the strains of the mother and father are reversed (Figure 1A). Multiple efficient software solutions are available to map short sequences from massive parallel sequencing to reference genomes, typically called aligners, and all of these report alignments as BAM files. In order to allow maximum flexibility Allelome.PRO is not dependent on a specific aligner, but rather requires one BAM file per biological replicate. The output of Allelome.PRO provides a categorization for each locus in the annotation file (Figure 1A). Furthermore, Allelome.PRO provides a BED file that allows a visual display of the data and can be viewed on any genome browser, such as the UCSC genome browser (http://genome.ucsc.edu, (47)).

### Allelome.PRO pipeline

The Allelome.PRO pipeline operates using a number of discrete sequential steps (Figure 1B). First reads from the aligned BAM files that overlap SNPs within the loci provided in the annotation file are extracted using filters that require BEDtools (46). This step limits analysis to reads informative for allelic analysis reducing the number of reads that need to be processed in subsequent steps and thereby improving the efficiency of the pipeline. The extent to which runtime is reduced depends on the number of SNPs, the proportion of the genome covered by the annotation file, and the genomic distribution of the sequencing data. Next, reads overlapping multiple SNPs are trimmed using a custom script, so that each read covers a single SNP and is counted only once, a necessary step for statistical analysis. SAMtools (45) is then used to generate a pileup file of reads at the SNP positions. Pileup files are used to calculate the total number of reads aligning to each allele. These numbers are summed up for all covered SNPs within each annotated locus separately for each of the four biological samples. A binomial test, implemented in R (44), is then used to assess the significance of deviation of the observed allelic biases from the expected 1:1 distribution for biallelic expression for each of the four samples. An allelic score is then calculated for each sample by negative logarithm transformation of the *P* value (–log_10_(*P*)) (29). Two scores are then calculated for each loci by comparing the four samples with each other, a parental bias and strain bias summary score. Loci are then assigned into allelic categories based on whether the allelic score is over the empirically derived false discovery rate (FDR) cutoff and a user defined allelic ratio. Calculation of the summary scores, the FDR and definition of the allelic ratio cutoff are described in detail below. The Allelome.PRO program can be downloaded at the following link: https://sourceforge.net/projects/allelomepro/.

### Validation of Allelome.PRO using RNA-seq and ChIP-seq of F1 MEFs

To validate the Allelome.PRO pipeline and define allelic expression and H3K4me3 enrichment in a pure cell type, we performed RNA-seq and ChIP-seq on female F1 MEFs derived from reciprocal crosses between the inbred mouse strains CAST/EiJ (CAST) and FVB/NJ (FVB). We performed two biological replicates from the forward and reverse cross to match the Allelome.PRO requirements. Sequencing reads were aligned to the GRCm38/mm10 genome using the STAR aligner (version 2.3.1z12) (37). For RNA-seq we performed strand-specific ribosomal depleted 100 bp paired-end RNA sequencing (see ‘Materials and Methods’ section). Ribosomal depletion of total RNA was chosen rather than polyA enrichment to allow analysis of intron located SNPs. On average we obtained 106.6 (±3.3) million total reads per biological replicate, 72% (±4%) of which were uniquely aligned. For ChIP-seq of H3K4me3 we applied 50 bp single-end sequencing and obtained 48.6 (±2.2) million total reads per biological replicate and 93% (±1%) uniquely aligned reads. For the Allelome.PRO run we downloaded SNP variant data from the Sanger institute (40) and extracted 20.4 million high quality SNPs between CAST and FVB (see ‘Materials and Methods’ section). We then used this data to validate and optimize the Allelome.PRO pipeline as described in the following sections.

### Calculation of the allelic score and false discovery rate

**Figure 2.**
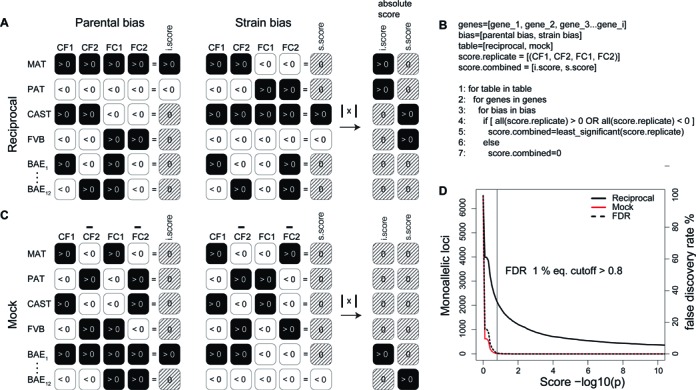
False discovery rate (FDR) allelic score cutoff based on mock analysis. (**A**) Two allelic scores were calculated for each annotated loci for each of the four samples, a parental bias score (MAT >0, black; PAT <0, white) and a strain bias score (CAST >0, black; FVB <0, white). The allelic score is defined as the negative logarithm of the binomial distribution of reads coming from one allele versus both alleles (–log_10_(*P*)). Reciprocal analysis was conducted to categorize allelic enrichment for each loci by comparing the parental bias scores (left) and strain bias scores (right) between the four samples. The allelic score patterns in the four samples for each allelic enrichment category are displayed: parental biased (MAT, PAT), strain-biased (CAST, FVB) and biallelic genes (BAE, only 2 of 12 possible biallelic combinations are displayed). A summary imprinted score (i.score) and strain-biased score (s.score) is calculated by comparing the four samples. If the bias is in the same direction for all four samples then the minimum score is taken, while if direction of bias is inconsistent for any of the four samples then the score is set to 0 (striped pattern). Each loci can have either an i.score value (imprinted) or an s.score value (strain biased), while the other score equals zero, or both the i.score and s.score equal 0 (biallelic). The absolute value of the i.score and s.score are calculated and then used for calculating the false discovery rate (FDR) in (D). (**B**) Pseudocode illustrating how the final allelic score (i.score or s.score) is derived from the allelic scores of the four biological replicates. (**C**) Mock analysis of parental bias and strain bias allelic scores to calculate i.scores and s.scores are conducted as for the reciprocal analysis in (A), except the scores of one sample from each cross are inverted. This results in the removal of parental bias and strain bias genes, which no longer have a consistent direction of bias and therefore have a score of 0. In contrast, 4 from 12 possible biallelic score combinations now have parental scores or strain bias scores in the same direction, resulting in a summary i.score or s.score value different from 0. These score values should be low compared to true allelic biases as they showed random deviations from a 0.5 ratio representing the technical and biological variation in the data. The absolute values of these mock scores are then compared to the values calculated in the reciprocal analysis to calculate the FDR in (D). (**D**) The false discovery rate (FDR) was estimated as the number of detected candidates with allelic biases (parental and strain bias) in the mock analysis, divided by the number of detected candidates with allelic biases in the reciprocal analysis. In this example RefSeq genes on the forward strand were analyzed in E12.5 mouse embryonic fibroblasts (MEFs) RNA-seq data using an FDR of 1%.

Two allelic scores, a parental bias score and a strain bias score, were calculated for each annotated region (RefSeq gene for RNA-seq, RefSeq gene TSS ± 2 kb for H3K4me3 ChIP-seq) in each F1 sample from two forward and two reverse FVB (F) and CAST (C) crosses (CF1, CF2, FC1, FC2). Previous approaches using a similar experimental design and statistical method calculated an allelic score for imprinted expression (imprinted score) from RNA-seq data for the 4 possible reciprocal comparisons (32,33). By calculating scores for the individual samples we were able to include SNPs covered in single samples that would be excluded in the reciprocal comparison approach, thereby increasing the power of the analysis. The parental bias score was calculated using summed maternal and paternal reads over SNPs per loci (MAT >0, PAT <0), and the strain bias score using summed strain 1 (CAST) and strain 2 (FVB) reads over SNPs per loci (CAST >0, FVB <0). To distinguish different categories of allelic enrichment we made two comparisons between the scores of the four samples, a parental bias and strain bias comparison as illustrated in the reciprocal tables (Figure 2A). Two summary scores were then calculated for each locus, a parental or imprinted score (i.score) and a strain bias score (s.score). If a consistent positive or negative bias was seen in all 4 samples, the lowest value was taken as the summary score, otherwise if the direction of the bias was not consistent the summary score was set to 0. Using this approach, each locus had a value for only one score (either the i.score or the s.score), while the other score equalled 0, or both scores were 0. That is, loci showed parental-specific enrichment (i.score >0 or <0, s.score = 0), strain-specific enrichment (i.score = 0, s.score >0 or <0) or non-enrichment (biallelic or non-informative, i.score = 0, s.score = 0) (Figure 2B the logic for the allelic score calculation). Finally, the absolute value (>0) of the i.score and s.score was calculated, a step necessary for the calculation of the false discovery rate (FDR) as described in the following section (Figure 2A and C).

There are 16 possible combinations of positive (>0) and negative (<0) allelic scores for the four samples, two of which show allelic biases in the same direction for parental bias, and two for strain bias (Figure 2A). It is expected by chance that 4 in 16 biallelic loci will also show an allelic bias in the same direction for all four samples, for either the parental or strain bias comparisons, leading to an i.score or s.score >0 or <0, although the allelic score should be lower than for true imprinted or strain bias loci as the allelic ratio should be close to 0.5. Therefore, we sought to reduce the number of false positives by setting a FDR based on the level of random allelic enrichment in our data empirically determined by mock analysis (Figure 2C). This approach was based on mock analysis of the four samples as reported earlier (32), but with several modifications. Previously, mock comparisons between samples of the same genotype were used to determine the FDR, as no difference in allelic expression is expected (32,33). Thus two mock comparisons are possible with four reciprocal comparisons. In contrast, we calculate a score for each sample rather than for the comparisons, enabling four scores to be used for the mock analysis and allowing the reciprocal and mock analysis to be performed in the same way. To perform mock analysis we negated the allelic scores in one biological replicate of each cross (CF2, FC2), and then performed the analysis in an identical manner as for the reciprocal analysis generating an i.score and s.score for each locus in the annotation (Figure 2C). Using this approach loci previously showing parental or strain bias (Figure 2A) now had an i.score and s.score of 0, while some biallelic loci (expected 4 in 12) now had an i.score or s.score >0 or <0 (Figure 2C). This mock analysis gave an estimate of the technical and biological variation in our data and was used to calculate the FDR. To calculate a single FDR for monoallelic enrichment we first pooled the absolute value of the i.score and s.score for all loci for both the reciprocal and mock analysis (Figure 2A and C). This differed from previous approaches that compared only parental allele bias to calculate the FDR (32,33), and increased the robustness of the FDR due to the larger number of strain bias loci compared to parental bias loci (∼20-fold higher in this study). The FDR (%) was calculated as the number of loci exceeding the score cutoff in the mock analysis, divided by the number of regions exceeding the score cutoff in the reciprocal analysis, multiplied by 100. For each run Allelome.PRO provides a plot showing the number of monoallelic loci at different score cutoffs in the reciprocal and the mock analysis as well as the corresponding FDR. A vertical line indicates the score cutoff at the user defined FDR, which in this study was 1% (Figure 2D).

### Empirical determination of an allelic enrichment cutoff

Defining allelic enrichment by an allelic score FDR cutoff alone can lead to artefacts, as biallelic genes can overcome the FDR cutoff if by chance all four samples share the same direction of bias and SNP coverage is high enough. Following this, we observed that if we analyzed our data with the FDR cutoff as the only filtering criteria, some highly expressed genes with small deviations from a biallelic ratio could produce scores over the FDR cutoff. Loci showing a minor allelic ratio bias are often not validated by independent methods (32), and even if validated there is no evidence that such minor biases are biologically meaningful. Therefore, we empirically determined an allelic ratio cutoff from our data based on known imprinted and strain bias genes, and used this to filter loci over the FDR cutoff to further reduce false positives.

**Figure 3.**
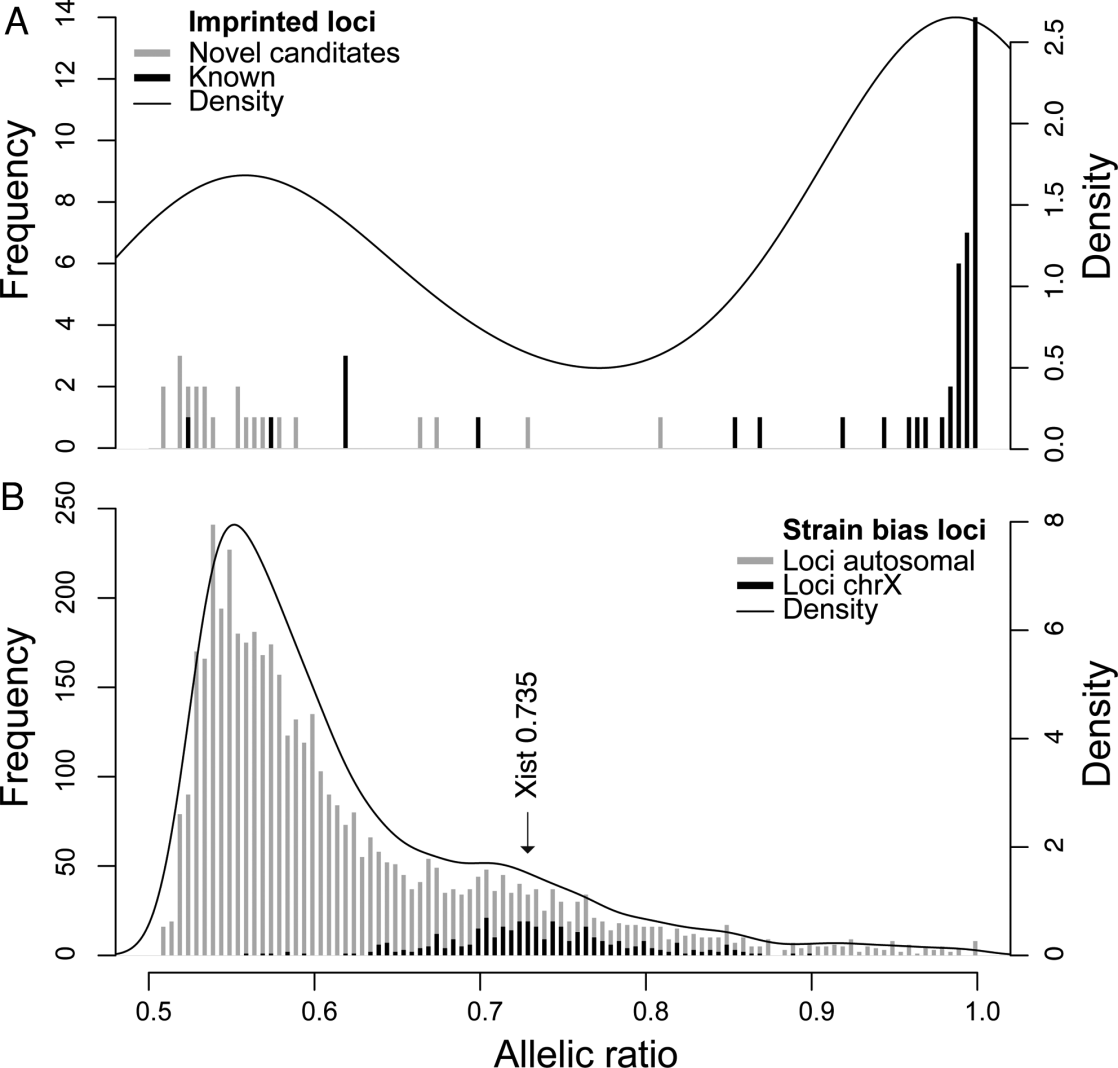
Setting the allelic ratio. (**A**) The allelic ratio distribution for the 65 parental bias genes with an i.score higher than the FDR cutoff in RNA-seq data from MEFs. Plotted are both a histogram in grey and a density curve for this distribution. The black bars overlapping the histogram indicate known imprinted genes. (**B**) The allelic ratio distribution for all strain-biased loci with an allelic score higher than the FDR cutoff in RNA-seq data from MEFs. Plotted are both a histogram in gray and a density curve for this distribution. The black bars overlapping the histogram indicate strain-biased loci on the X chromosome. Densities were estimated using a Gaussian kernel function calculated in R.

In order to determine a biologically relevant allelic ratio cutoff we first plotted the distribution of allelic ratios for all 65 genes classified as imprinted by the FDR cutoff in analysis of our RNA-seq data (Figure 3A). Of these 43 have been reported to show imprinted expression previously (29,42,43). Notably, a biphasic distribution was obtained with most known imprinted genes showing allelic ratios >0.85, and most of the novel imprinted genes identified by our RNA-seq showing much lower allelic ratios. Six known imprinted genes (*H13, Gnas, Inpp5f, Phactr2, Cobl, Trappc9*) were also clustered in this low ratio group. However, these are all genes with reported tissue-specific imprinted expression in a tissue other than MEFs (27,48–52). No novel imprinted gene was identified in the RefSeq annotation with this allelic ratio cut-off.

To determine an appropriate FDR cutoff for genes with a strain biased expression pattern, we made use of known skewed X-inactivation in our MEFs. This is a well-documented effect in female cells from crosses between *M. musculus domesticus* (FVB) and *M. musculus castaneus* (CAST) that results in the predominant inactivation of the FVB derived X-chromosome (13,14). The allelic ratio distribution of genes on autosomes showed a prominent peak between 0.5 and 0.6, close to a biallelic expression ratio (Figure 3B). However, we also noted a shoulder peak around 0.7, which followed the distribution of the X-linked genes (Figure 3B, black bars). Following this, genes on the X-chromosome showed a mean allelic ratio of 0.735 in our analysis. The majority of X-linked genes showing a significant strain bias over a ratio of 0.7 (>85%). Therefore, in order to have a single allelic cutoff for strain biased and parental biased genes and to distinguish allelic bias from biallelic expression, we used an allelic ratio >0.7 cutoff together with a 1% FDR cutoff for further analyses. At an allelic ratio cutoff of 0.7, two novel candidate imprinted genes were detected in addition to the 37 known imprinted genes detected with an allelic ratio cutoff of 0.85. These genes were not detected in a previous study of MEFs (Table 1) (53), and were not validated by differential H3K4me3 analysis (Table 2), indicating that they were false positives. This indicates that a lower parental bias allelic ratio cutoff is acceptable when novel candidates are subject to independent validation.

**Table 1.** RefSeq genes showing imprinted expression in MEFs detected by Allelome.PRO

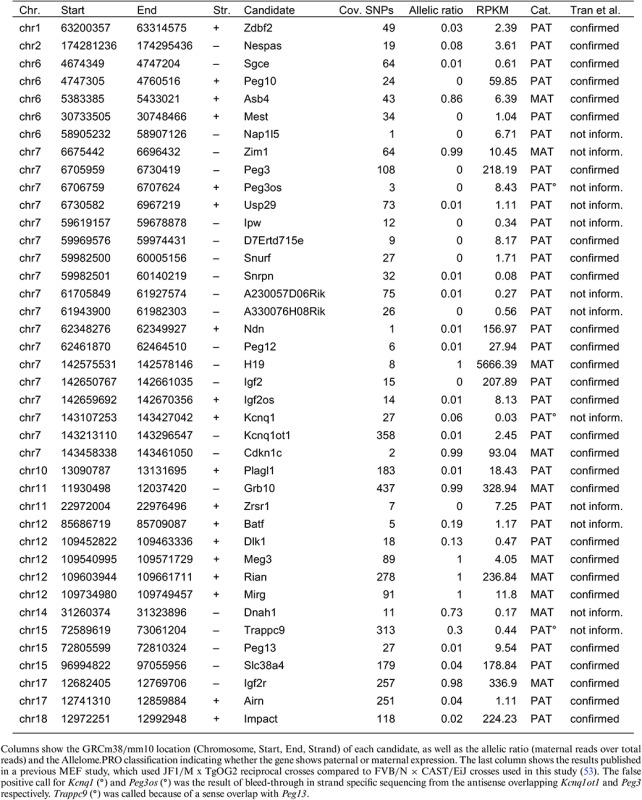									

**Table 2.** H3K4me3 ChIPseq data results confirm the RNA-seq allelome

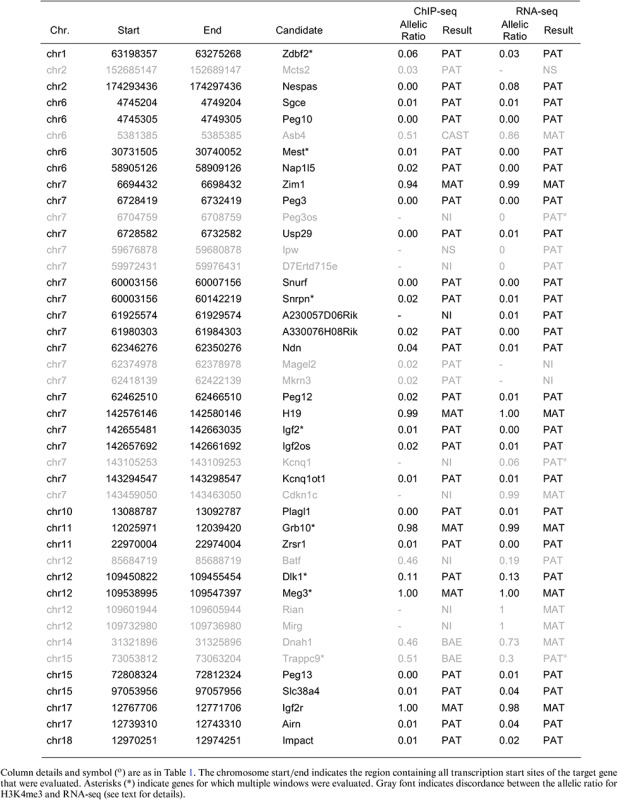							

### Genome-wide allele-specific expression in MEFs

Previous studies analyzed RNA-seq, ChIP-seq or DNA methylation-seq to detect either parental or strain specific allelic enrichment, but none defined the allelic enrichment status, or allelome, of all annotated loci in a given cell type or tissue. In order to do this, we defined biallelic loci as those loci not identified as showing a parental or strain bias, but with enough SNP coverage to theoretically overcome the allelic score FDR at an allelic ratio cutoff of 0.7. Those loci with a lower SNP coverage (non-expressed or very lowly expressed genes) were defined as non-informative, while the final category included loci with no SNPs. In summary, by introducing an allelic ratio cutoff in combination with an allelic score cutoff, Allelome.PRO was able to categorize all loci in an annotation into 7 categories: maternal bias (MAT), paternal bias (PAT), strain 1 bias (CAST), strain 2 bias (FVB), biallelic (BAE), non-informative (NI) and no SNP (NS).

**Figure 4.**
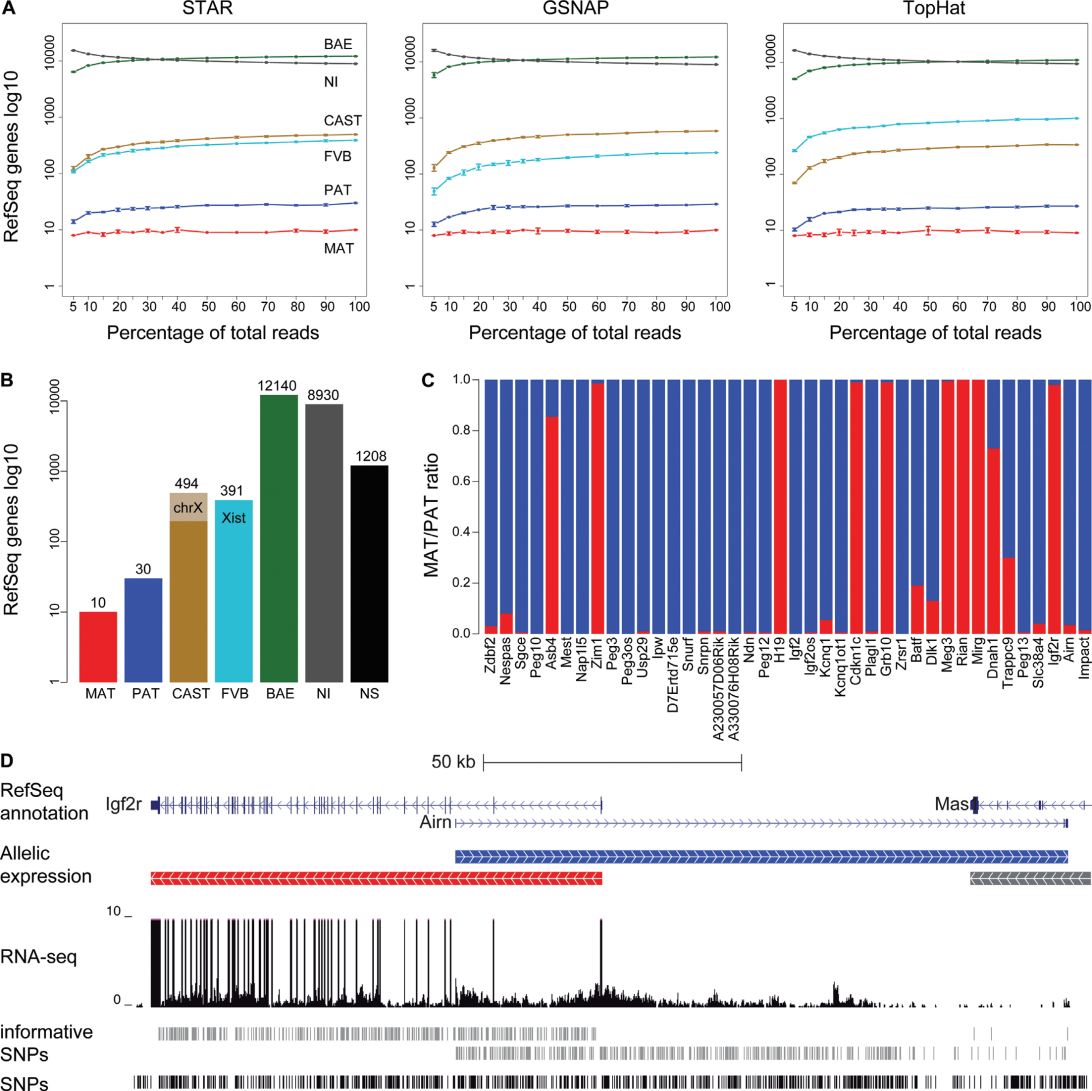
Allelome defined in MEFs using RNA-seq. (**A**) Saturation curves showing the Allelome.PRO results for different samplings of the total RNA-seq reads from MEFs for three different aligners, STAR, GSNAP and TopHat (from left to right). Reads were sampled from total uniquely aligned reads in three technical replicates (STAR: 77.47 ± 1.89, GSAP: 76.89 ± 1.94, TopHat: 94.47 ± 2.02 millions of reads per replicate). The six curves in each plot represent the categories listed in Figure 1 except the ‘No SNP’ category, which is omitted. The curves for imprinted genes (blue, red) show saturation at low sample rates and little differences between the three aligners. The curves for strain-biased genes (brown, turquoise) show an increase of strain-biased genes with increasing read number, although the slope decreases with higher sample rates it does not plateau. The three aligners detect different numbers of strain bias genes, with STAR and GSNAP detecting more CAST than FVB biased genes, while TopHat detects more FVB than CAST strain bias genes. All aligners show an increase in the number of biallelic genes detected, and a decrease in the number of non-informative genes with increasing number of sequencing reads. (**B**) Categorization of RefSeq genes as produced by Allelome.PRO for strand-specific RNA-seq data of MEFs. Genes were categorized into seven categories, as listed in Figure 1 with numbers given above. The pale brown bar shows the amount of CAST strain-biased genes on chromosome X. *Xist* shows a strain bias towards the FVB allele as indicated on the turquoise bar. (**C**) The imprinted genes from (B) in more detail. The ratio between maternal and paternal allele is illustrated as red and blue bars. Genes were sorted by chromosome number and genomic location and gene names are given on the x-axis. This Figure is also part of the Allelome.PRO output. (**D**) Ribosomal depletion followed by 100bp paired end deep sequencing allows the detection of SNPs within the introns of protein coding genes and lowly expressed long non-coding (lnc) RNAs. UCSC genome browser screenshot showing data on the protein-coding gene *Igf2r* and the long non-coding (lnc) RNA *Airn* in MEFs. The tracks depict (from top to bottom): RefSeq genes, Allelome.PRO allelic expression categorization, RNA-seq aligned reads, informative SNPs on the forward and reverse strand in grey, and total SNPs in black.

Next, we investigated how many RNA-seq sequencing reads were necessary to categorize allelic data, and which alignment program produced the best results with RNA-seq data. The saturation curves for the STAR aligner showed saturation for the number of imprinted genes (red and blue) already at low sampling rates (10–20%, or 10–20 million reads per replicate) (Figure 4A, left) (37). In contrast, the numbers of strain-biased (brown and turquoise) and biallelic genes (green) continued to increase with increasing number of reads, although the slope decreased with higher sample rates indicating the data was near saturation. With increased sequencing depth the number of biallelically expressed genes increased in parallel with a decrease in the number of non-informative genes. Saturation curves were also produced from the data aligned with GSNAP and TopHat (Figure 4A, middle and right, respectively) (38,39). The saturation curves were broadly similar for all three aligners, with the exception of strain-biased genes. Both STAR and GSNAP detected more CAST than FVB strain bias genes (494 CAST versus 391 FVB (STAR) and 583 CAST versus 240 FVB (GSNAP)), but in contrast TopHat detected more FVB than CAST strain bias genes (338 CAST versus 1009 FVB). This is likely due to an alignment bias, as the FVB strain is more closely related to the C57BL/6J reference strain than CAST, and therefore TopHat may have difficultly aligning some CAST reads leading to false positive FVB strain bias genes and the failure to detect some CAST bias genes. We observed that STAR aligned more reads over SNP positions than GSNAP or TopHat, which, combined with the much shorter runtime, convinced us to use STAR for further analysis. Still, one of our main concerns in choosing STAR over GSNAP was that STAR does not offer an option for SNP-sensitive analysis like GSNAP. However, when we correlated allelic ratios determined by GSNAP and by STAR they showed very high correlation (*R*^2^ = 0.99), indicating that alignment biases did not affect the overall results.

The results for the Allelome.PRO run with STAR aligned data showed 40 imprinted genes, 10 of which were maternally expressed while 30 showed paternal expression. Furthermore, we detected 494 CAST bias (299 on chromosome X) and 391 FVB bias genes (three on chromosome X), confirming the X-inactivation between these strains. Of the remaining genes, 12140 were classified as showing biallelic expression, 8930 as non-informative and 1208 could not be assessed as they contained no SNP (Figure 4B). The allelic ratios of the detected imprinted genes are displayed in Figure 4C with detailed information including genomic location, number of covered SNPs, and allelic ratio given in Table 1. Additionally, details for all informative SNPs over detected imprinted genes is given in Supplementary Table S1, and the full table including all informative genes is available from the Gene Expression Omnibus (GEO, accession number GSE69168). Only 31 of the 123 known imprinted genes were detected as imprinted (Supplementary Table S2). In most cases, this was likely due to tissue-specific imprinted expression where the genes were called non-informative (not expressed) or biallelic, although six genes could not be assessed for imprinted expression due to strain bias, and seven genes were not assessed because they were not included in the RefSeq annotation that we used. Our results showed a high level of agreement with a previous RNA-seq study conducted in MEFs from a JF1/M × TgOG2 reciprocal crosses, with 27 of 32 reported imprinted genes detected (Table 1) (53). Of the five genes that we did not detect, one had no SNP in our cross (*Nnat*), two were not part of the RefSeq annotation that we used (*AK050713* and *Rtl1as*), and we excluded one from our annotation due to its small size (*AF357425*). However, using Allelome.PRO with sliding window annotations (2, 4, 6 and 8 kb) we could confirm imprinted expression of *AK050713*, *Rtl1as* and *AF357425* (data not shown). The fifth candidate, *Blcap*, was categorized as biallelically expressed in our data, which was in agreement with reports that this gene only exhibits imprinted expression in brain (54). We detected 13 imprinted gene candidates by RNA-seq in our study that were not detected in a previous study of MEFs (53). Two were probable false positives due to overlap with other imprinted genes: *Kcnq1* due to anti-sense overlap with *Kcnq1ot1* and the incomplete strand-specificity of our sequencing technique, and *Trappc9* due to sense overlap with *Peg13*. *Kcnq1* and *Trappc9* were lowly expressed compared to their overlapping genes, and visual inspection of the genome browser revealed that these long genes showed an increased signal in the overlap region with the shorter *Kcnq1ot1* and *Peg13*, further indicating that this signal from this overlapping region was responsible for the probable false positive call. We called *Dlk1* as paternally imprinted, while the previous study classified it as paternally biased and did not include it in their final list (53). The remaining 10 imprinted gene candidates that we detected were characterized in the previous study as either lacking SNPs, being non-expressed or low expressed, or no data was presented (53). This indicates the increased sensitivity of our method due to the large number of SNPs used, our ability to detect SNPs in introns due to the use of total RNA-seq, and the Allelome.PRO approach of summing all covered SNPs within a gene, all of which together enabled us to detect imprinted expression of lowly expressed genes. Similarly, using all SNPs covered in at least one replicate, and summing up all SNPs within a gene, enabled us to also call lowly expressed long non-coding (lnc) RNAs with high confidence. This was illustrated by the example of *Igf2r* and *Airn* (Figure 4D). Coverage across the *Airn* gene body was much lower than coverage of *Igf2r* exons, but due to the large number of available SNPs (grey bottom track, informative SNPs) typical for a non-coding gene, the *Airn* lncRNA was still confidently called as showing paternal expression.

In summary, using Allelome.PRO to analyze RNA-seq data we confirmed previously reported imprinted genes in MEFs and detected additional genes, most of which were previously reported to show imprinted expression in another tissue. We also detected strain biased genes, including X-linked genes confirming a known X-chromosome inactivation bias, as well as classifying biallelic expressed and non-informative genes, thus defining for the first time the entire allelome of a tissue.

### Validation of allele specific expression by H3K4me3 ChIP-seq

**Figure 5.**
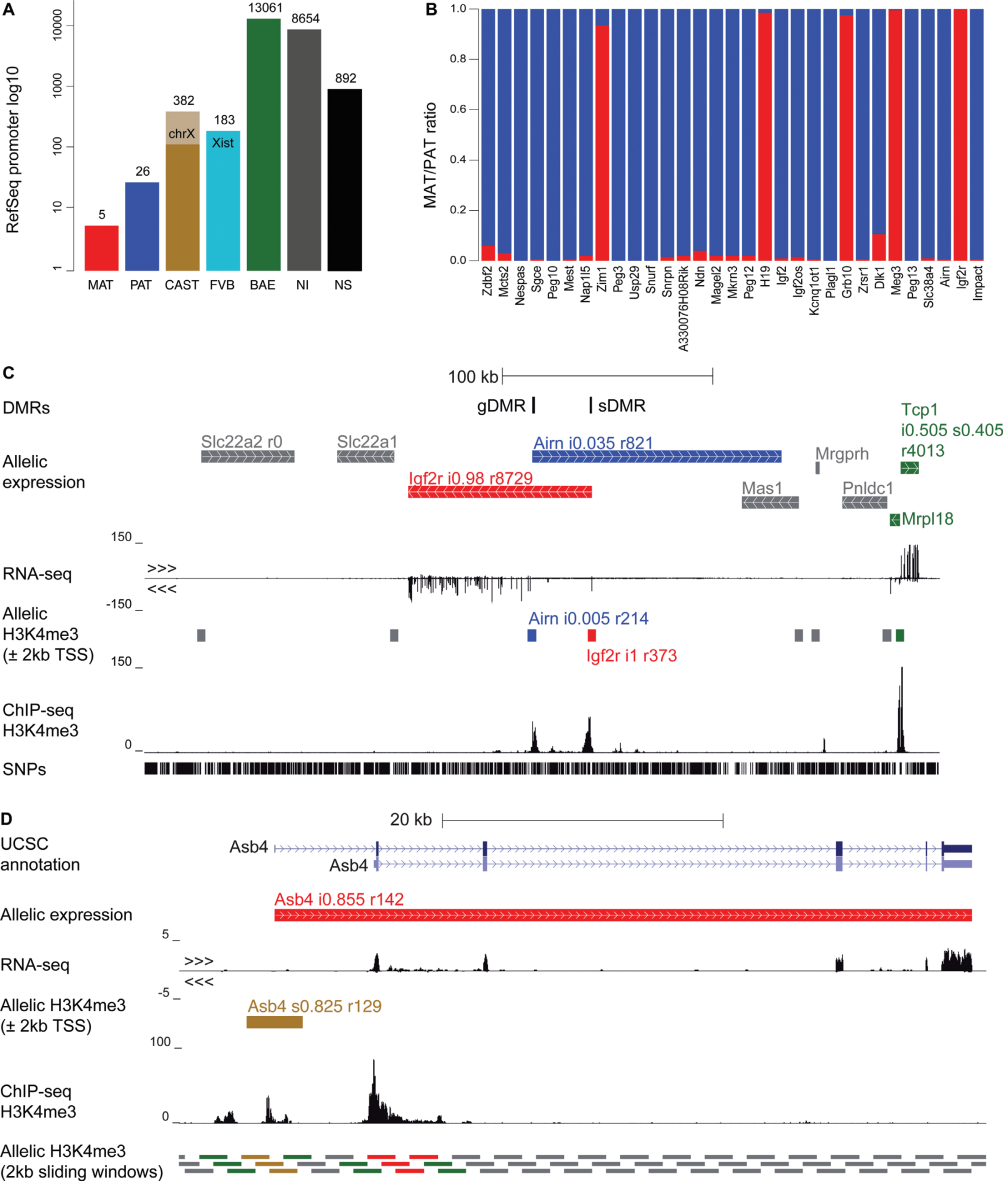
Allelome validated using ChIP-seq H3K4me3. (**A**) Categorization of RefSeq promoter regions (±2 kb windows over RefSeq gene transcription start sites) as produced by the Allelome.PRO for H3K4me3 ChIP sequencing data of MEFs. The CAST strain bias on chromosome X is seen as well as the categorization of *Xist* as showing a FVB strain bias. (**B**) The maternal/paternal ratio is shown as red/blue bars for the imprinted genes from (A). Promoter windows are named after their respective genes and sorted as in Figure 4C. (**C**) Allelic expression in the *Igf2r* cluster is validated by differential H3K4me3 enrichment in MEFs. UCSC genome browser screenshot showing the *Igf2r* imprinted gene cluster and adjacent genes together with the Allelome.PRO output for RNA-seq and ChIP-seq. The tracks depict (from top to bottom): gametic and somatic differentially DNA methylated regions (gDMRs and sDMRs), Allelome.PRO allelic expression categorization, strand-specific RNA-seq, Allelome.PRO H3K4me3 allelic enrichment categorization, H3K4me3 ChIP-seq, and total SNPs in black. (**D**) Sliding windows detect differential H3K4me3 peaks outside annotated transcription start sites. Allelome.PRO detected maternal expression of *Asb4* from RNA-seq data using the RefSeq annotation, but a CAST strain bias was detected by Allelome.PRO analysis of H3K4me3 ChIP-seq data using the RefSeq promoter annotation. However, analysing the ChIP-seq data instead with a 2 kb sliding window annotation revealed maternal H3K4me3 enrichment over the promoter of a shorter isoform of Asb4 that was annotated by UCSC and appeared from RNA-seq data to be the predominant isoform in MEFs. UCSC genome browser screenshot showing (from top to bottom) the UCSC gene annotation, Allelome.PRO allelic expression categorization from RefSeq, strand specific RNA-seq, Allelome.PRO H3K4me3 allelic enrichment categorization using RefSeq annotation, H3K4me3 ChIP-seq, and Allelome,PRO H3K4me3 allelic enrichment categorization using a 2 kb sliding window annotation.

Previously most RNA-seq studies investigating imprinted expression validated their results using methods that assay allelic expression using a single SNP in cDNA, for example, by pyrosequencing (32), Sanger sequencing (29), or Sequenom assays (31,53). Here, we validated our RNA-seq results using differential enrichment of the active H3K4me3 mark over promoters detected by Allelome.PRO using multiple SNPs from ChIP-seq data. Differential enrichment or H3K4me3, H3K27ac or H3K36me3 was used before as a proxy for imprinted expression of some imprinted genes, but not as a general validation of imprinted or allelic expression (30,55). Here we used 4kb windows surrounding the TSS of RefSeq genes as an annotation file for Allellome.PRO to analyze ChIP-seq data for H3K4me3 marks in MEFs. If a gene had multiple isoforms with different start sites, SNPs from all sites were combined, as each gene was treated as a single locus in this analysis. Using this approach our results were broadly similar to the RNA-seq results. We found 31 parental specific promoter marks, 5 maternal and 26 paternal (Figure 5A and B). The details of the informative SNPs for these genes are shown in Supplementary Table S1. 382 CAST specific promoter marks (272 on chromosome X) and 183 FVB specific promoter marks (1 on chromosome X) were found, confirming the X-inactivation bias seen by RNA-seq (Figure 5A). 13061 promoter regions were classified as showing biallelic marks and 8654 regions were non-informative, while 892 regions were not assessed because they contained no SNP (Figure 5A). A table including SNP details for all informative promoters is available from GEO (accession number GSE69168).

A high level of agreement was seen between imprinted genes detected in MEFs by RNA-seq and H3K4me3 ChIP-seq using Allelome.PRO. In total 43 genes were detected as showing parental specific expression and/or parental specific histone marks (Table 2). A comparison between the RNA-seq and ChIP-seq results showed that 28 out of 40 genes called as showing imprinted expression by RNA-seq also had differential enrichment of H3K4me3 over their promoter. Five of 12 genes not confirmed by ChIP-seq were found to show imprinted expression in MEFs by RNA-seq in an independent study, indicating that they do show imprinted expression in this tissue (Tables 1 and 2) (53). One showed a CAST bias in H3K4me3 enrichment (*Asb4*), while 4 others had non-informative H3K4me3 data (*D7Ertd715e*, *Cdkn1c*, *Rian* and *Mirg*). Seven of 12 genes not confirmed by ChIP-seq were also not found in a previous study of MEFs (Tables 1 and 2) (53). Two of these genes were probable artefacts caused by sense and anti-sense transcriptional overlap by other imprinted genes as mentioned previously (*Kcnq1* and *Trappc9*), demonstrating the value of differential H3K4me3 enrichment assays to resolve such issues. *Peg3os* also shows an antisense overlap with the highly expressed paternal imprinted gene Peg3, and was not confirmed by ChIP-seq, so it cannot be excluded that it is also not a false positive. Two other genes were the only novel candidate imprinted genes that we detected by the RNA-seq analysis (*Batf* and *Dnah1*). As mentioned previously, these genes were the only 2 of 40 imprinted expression candidates detected by RNA-seq that were not detected with the higher allelic ratio cutoff of 0.85, and this together with the lack of validation by H3K4me3 differential enrichment indicates that they are false positives. The remaining two genes not detected in the previous study of MEFs had high allelic ratios in our RNA-seq, but had non-informative ChIP-seq results (*A230057D06Rik*) or no SNP in the assayed region (*Ipw*). The three genes detected by ChIP-seq, but not by RNA-seq in our study were all known imprinted genes with high allelic ratios, and either had no SNP in the gene body (*Mcts2*), or had a non-informative RNA-seq result (*Magel2* and *Mkrn3*). In the previous study of MEFs, *Mcts2*, a 795bp single exon gene, was also reported to have no SNP, while *Magel2* and *Mkrn3* were described as not being expressed (53). We found *Magel2* to be lowly expressed, resulting in a non-informative result by RNA-seq. However, in our data *Mkrn3* was highly expressed, but three of four SNPs were excluded due to overlap with pseudogenes leading to the non-informative RNA-seq call. In summary, 33 out of 43 RefSeq genes that we detected as showing imprinted expression were found either by RNA-seq and ChIP-seq, or by RNA-seq and a previous study of MEFs, or in all three datasets, making these high confidence imprinted genes in MEFs. Additionally, six genes were found to be imprinted in either RNA-seq or ChIP-seq data, but not in the complementary dataset due to lack of SNPs (two genes) or a non-informative result (3 genes). The remaining five genes were excluded as probable false positives. Thus, 38 RefSeq genes were identified as showing imprinted expression in MEFs.

**Figure 6.**
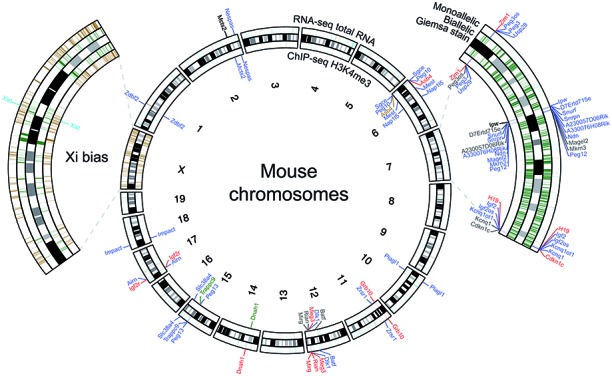
Allelome defined in MEFs. Allelome.PRO results are shown for all chromosomes on a circular representation. The tracks of the main plot in the middle are (from outside to inside): Mono-allelically expressed genes (i.e. strain-biased and imprinted), Giemsa chromosome staining (47) and genes showing allele specific H3K4me3 peaks in their promoter regions based on ChIP-seq. The two enlarged chromosomes were selected to demonstrate both the large amount of imprinted genes on chromosome 7 as well as the strain-biased X inactivation. In addition to the two outmost tracks showing mono-allelic genes also biallelic genes are shown in green for these two chromosomes (inner tracks).

To examine the results generated by Allelome.PRO in detail, we used the well-characterized *Igf2r* imprinted gene cluster (Figure 5C). The RNA-seq and ChIP-seq results confirmed that *Igf2r* was only expressed from the maternal allele, whereas the macro lncRNA *Airn* was only expressed from the paternal allele. The extra-embryonic-lineage specific imprinted genes *Slc22a2* and *Slc22a3* are not expressed in MEFs and were therefore classified as non-informative in both analyses, as were *Mas1*, *Mrgprh*, and *Pnldc1*. *Tcp1* and *Mrpl18* showed biallelic expression and biallelic H3K4me3 marks. Overall, Allelome.PRO analysis for RNA-seq data showed the expected pattern for the imprinted *Igf2r* cluster that was confirmed by the Allelome.PRO H3K4me3 ChIP-seq analysis. As mentioned above, one example where the ChIP-seq and RNA-seq analyses disagreed was the maternally expressed gene *Asb4* (Figure 5D). The RefSeq annotation only contained the long isoform of *Asb4*, which showed a strain specific H3K4me3 peak at its transcription start site. However, Allelome.PRO run using a sliding window annotation for the same ChIP-seq data revealed the presence of a maternal peak at the start site of an UCSC-annotated shorter isoform of *Asb4*, confirming maternal expression in the RNA-seq results. Overall, ChIP-seq results showed agreement with RNA-seq results in 18180 (84%) of 21649 cases where at least one SNP was present in both analyses (Figure 6). Strain-biased expression showed the lowest validation rate with only 257 (29%) of 879 cases validated by ChIP-seq. Biallelic expression was confirmed for 10 922 (90%) of 12 058 candidates and 6973 genes were non-informative in both analyses.

## DISCUSSION

Since the development of high-throughput sequencing technologies a number of studies have sought to detect imprinted expression from RNA-seq data from various tissues using a variety of experimental and analysis approaches (26,27,29,31,32,56,57). Additionally, detection of differential allelic expression has potential for the mapping of *cis*-regulatory elements, so-called *cis* expression quantitative loci or *cis*-eQTLs (40,58–61). Furthermore, differential allelic expression analysis has been employed as a tool for studying alternative mRNA processing (12). We developed Allelome.PRO as a user-friendly and efficient tool to capture the genome wide state of differential allelic features, thus providing a single tool to aid in these different applications.

### Allelome.PRO provides a robust and sensitive tool to detect allelic enrichment

Detection of imprinted expression requires biological replicates and an empirical method to set the FDR from the data in order to take account of the biological and technical variation in the system and minimize the chance of false positives (32). On the other hand the experimental setup and analysis pipeline has to be sensitive enough to detect all imprinted genes in a given tissue. Additionally, previous analytical pipelines require a high level of bioinformatic expertise to implement. In contrast, Allelome.PRO is an efficient package that can function with minimal computer resources (tested on iMac 5.1, 3Gb RAM, Dual-core 2.16 GHz processor), and that based on a limited number of user-set parameters will automatically process the aligned sequencing data provided to set the FDR, categorize allelic enrichment of all loci in an annotation file, and output the analyzed data both as a table, as summary graphs and as a BED file that can be uploaded and viewed on a genome browser (detailed in the manual, Supplementary material).

Allelome.PRO was developed based on the approach taken by Babak and colleagues to detect imprinted expression from RNA-seq data (32,33). Following this we used tissue from F1 offspring from two reciprocal crosses (four samples), combining allelic counts of multiple SNPs within candidate loci, and calculated allelic scores based on the binomial distribution. In contrast to previous approaches (32,33), who calculated the allelic score based on the four possible reciprocal comparisons between the samples, we calculated the allelic score for each of the four samples separately and detected allelic bias as loci where the direction of the bias was the same in all four samples. This allowed us to include all SNPs covered in at least one sample increasing the sensitivity of our method. Additionally, this approach of calculating allelic scores for each sample could be adapted to include more than four samples to increase statistical confidence in situations where reciprocal crosses from inbred strains are not available but SNPs are well-characterized, as is the case in humans. Outbred species where SNPs have not been characterized could also be examined if a SNP calling program such as SAMtools or GATK is first used to call SNPs *de novo* (45,62). To maximize sensitivity to detect allelic expression, we performed RNA-seq using rRNA depleted total RNA, which provided increased coverage of SNP-rich intronic regions. In order to count all reads covering a SNP we trimmed reads covering multiple SNPs so that only a single SNP was counted, rather than excluding SNPs by their distance to other SNPs as done previously (32,33). All of these steps together helped increase the sensitivity of our approach.

In order to empirically calculate the FDR based on the data, previous approaches used the two possible mock comparisons between F1 samples of the same genotype, a comparison that should lack allelic differences (32,33). In contrast, we did mock analysis by inverting the scores of two biological replicates and comparing the four samples, exactly as for calculating the normal score, thereby using the variation in biallelic genes to calculate the FDR. Additionally, in contrast to previous approaches that calculated the FDR by comparing the imprinted score for reciprocal and mock comparisons alone (32,33), we included both imprinted and strain bias scores in our reciprocal to mock comparisons to calculate a single allelic FDR cutoff. Basing an FDR cutoff on imprinted genes alone does not allow a low cutoff to be set due to the limited number of imprinted genes. For example, with the 40 imprinted genes detected by RNA-seq in this study our FDR cutoff of 1% would not be reached until 0 genes are detected in the mock comparisons, making an effective FDR cutoff of 0%. Therefore, by including the several hundred strain bias genes (885 in our study) in the FDR calculation we are able to set a lower FDR cutoff than in previous studies (32,33), increasing the robustness of our pipeline.

A key innovation in the Allelome.PRO pipeline compared the approach taken by Babak *et al*. (32,33), is the introduction of an allelic ratio cutoff, in addition to the allelic score FDR cutoff, to further reduce false positives. One of the caveats of using the binomial distribution is that even small deviations from a 0.5 ratio could result in a score over the FDR if the amount of reads is high enough. As small differences in the ratio are likely due to chance, and even if true, are unlikely to cause a phenotype, we defined an allelic ratio cutoff to separate true allelic biases from stochastic variations. The introduction of an allelic ratio threshold was also proposed by Wang and Clark (63), who suggested a 0.65 ratio based on their experience that imprinted candidates below this ratio could rarely be validated. Other allelic ratios thresholds used in previous studies range from 0.6 (64), to 0.8 (53). Based on the allelic ratio distribution of known imprinted and strain-biased genes in our RNA-seq data from MEFs, we chose 0.7 as an allelic ratio cutoff. Above this allelic ratio cutoff, 95% of imprinted gene candidates were known imprinted genes, and 85% of X-linked genes that showed a strain bias over the FDR cutoff were included. The introduction of an allelic ratio cutoff allows imprinted and strain bias loci to be distinguished from biallelic loci. In order to classify all annotated loci in an annotation file we defined non-informative genes as those with less SNP coverage than theoretically necessary to overcome the allelic ratio cutoff. This enabled Allelome.PRO to classify all loci as either parental biased (imprinted), strain biased, biallelic, non-informative or lacking SNPs. Therefore, in contrast to previous approaches that sought to detect imprinted expression from RNA-seq data, we provide a tool to categorize the entire allelic expression of all annotated loci in a given tissue, allowing other allelic expression types to be identified and investigated. Moreover, Allelome.PRO is flexible in that it will function with any annotation and sequencing data provided, as demonstrated in this study where both RNA-seq and H3K4me3 ChIP-seq were analyzed and showed a high correlation with each other.

To further test the robustness of the Allelome.PRO pipeline we simulated different rates of sequencing error in our MEF RNA-seq data in the region surrounding the *Igf2r* imprinted gene cluster, and assessed the effect on the Allelome.PRO results (Supplementary Figure S1). We found that *Airn* and *Igf2r* were correctly called imprinted even at error rates of 10 and 15% when the number of aligned reads was greatly reduced. The biallelic gene *Mrpl18* also remained biallelic at a 10% error rate before becoming non-informative at a 15% error rate. However, at these higher error rates the biallelic gene *Tcp1* gene became FVB strain biased, perhaps because FVB has less SNPs with the reference genome compared to CAST, and therefore more FVB reads may align, indicating that strain biased calls may be affected by high rates of sequencing errors (Supplementary Figure S1). To test the impact of experimental error in our method on the Allelome.PRO results we mixed *in silico* reads from FVB and CAST adult heart to create four pools that mimicked two forward and reverse crosses required for Allelome.PRO (detailed in methods). The allelic ratios showed the expected Gaussian distribution centered around 0.5, with deviations from the mean likely due to strain biased genes (Supplementary Figure S2A). As no imprinted genes are expected in such a mixing experiment, we defined the FDR as the percentage of informative genes called imprinted. The FDR was low (0.15%) and could be further decreased by increasing the allelic ratio cutoff or minread parameter (Supplementary Figure S2B), although increasing the minread parameter also decreased the number of informative genes, thus reducing sensitivity (Supplementary Figure S2C). At the 0.7 allelic ratio cutoff and minread 1 settings used in this manuscript, the FDR was reduced to 0.01%. In summary, analysis of the effects of sequencing errors and general experimental errors show the robustness of the Allelome.PRO pipeline in defining allelic expression.

### Allelome.PRO defines the MEF Allelome using RNA-seq and ChIP-seq

We detected allele specific expression in MEFs using Allelome.PRO to analyze RNA-seq data, and then validated the results using Allelome.PRO analysis of H3K4me3 ChIP-seq data and by comparison to a previous study of imprinted expression in MEFs (53). We detected 40 genes showing imprinted expression from RNA-seq data using the RefSeq annotation, and 31 genes from ChIP-seq data using 2 kb ± RefSeq TSS, giving 43 genes that were detected by one or both methods. Twenty eight of the 40 genes that were detected by RNA-seq were validated by differential H3K4me3 enrichment over their promoters (70%), and a further five by detection in a previous study of MEFs (53). Another six genes were detected only by RNA-seq or ChIP-seq, but all were known imprinted genes and had high allelic ratios (0.03 or less), making it likely that they also show imprinted expression in MEFs. In addition to these 38 genes, we were able to detect three of five additional imprinted genes reported by a previous study of MEFs (53) using a sliding window annotation to assay our RNA-seq data. We did not initially detect these genes because they are not in the RefSeq annotation or were excluded because of small size. This indicates that we may detect a limited number of additional imprinted genes if we use other annotations in addition to RefSeq. More imprinted genes were detected by RNA-seq than ChIP-seq indicating it was more sensitive, although 5 of 40 genes appeared to be false positives. In contrast, there was no indication that any of the 31 genes detected by ChIP-seq were falsely called.

Some cases where ChIP-seq did not confirm RNA-seq may be due to incomplete annotation by RefSeq. This can arise if neighbouring imprinted lncRNAs are in fact continuous transcripts. If this is not annotated then multiple genes would be called by RNA-seq, and only 1 by ChIP-seq, due to the single promoter. For example, the neighbouring Riken transcripts A330076H08Rik and A230057D06Rik showed this pattern, with both being called imprinted by RNA-seq and being expressed at a similar low level, but only A330076H08Rik was also detected by ChIP-seq. Genes can have alterative start sites that may not be annotated by RefSeq, which would affect our ChIP-seq analysis based on windows around the RefSeq TSS. This was demonstrated by the example of *Asb4*, which was detected as imprinted in the RNA-seq analysis, but not validated by ChIP-seq analysis using a RefSeq promoter annotation. However, using a sliding window annotation we could validate imprinted expression of *Asb4*, finding differential H3K4me3 enrichment at an alternative promoter annotated in the UCSC gene track (47). Therefore, the validation rate of RNA-seq results by H3K4me3 ChIP-seq could be increased by using a more extensive annotation than RefSeq, such as UCSC genes, using peak calling programs, or by using the unbiased sliding windows approach.

Of the five genes considered false positive, three overlapped known imprinted genes: *Kcnq1* and *Peg3os* were called because of incomplete strand-specificity leading to bleed-through from the antisense overlapping *Kcnq1ot1* and *Peg3* respectively, while *Trappc9* was called because of sense overlap with *Peg3*. All 3 of these genes were lowly expressed compared with the overlapping imprinted genes (RPKM <5% the overlapping gene, Table 1), and were not confirmed by H3K4me3 ChIP-seq analysis. We generated strand-specificity using a method based on dUTP incorporation into second strand cDNA synthesis and subsequent uracil-N-glycosylase degradation (35), where bleed-through may occur due to incomplete degradation of the second strand or spurious second strand synthesis by reverse transcriptase (65). The remaining two genes (*Batf* and *Dnah*) were novel imprinted candidates, and were considered false positives because they had lower allelic ratios than the other imprinted genes detected by RNA-seq, and they were not validated by ChIP-seq or by being previously detected in other studies. These two genes were relatively lowly expressed and could be excluded if we adjusted the minread parameter in the Allelome.PRO pipeline. By increasing this parameter to only include SNPs covered by at least three reads (instead of 1 read) we observed that *Batf* and *Dnah* were then called non-informative. Additionally, *Kcnq1* was also then categorized as non-informative, indicating that increasing this parameter also made the pipeline more resistant to false calls due to bleed-through from the opposite strand. Therefore, we suggest that increasing the minreads parameter should be used to decrease the number of false-positives due to low coverage where no additional validation method, such as ChIP-seq, is available.

Allelome.PRO categorizes the allelic enrichment status of all loci in an annotation in a given tissue, enabling other categories in addition to imprinted genes to be investigated. In MEFs by analysis of RNA-seq data we found 885 genes showing strain bias expression, 34% were CAST biased X-linked genes due to a known bias in X-inactivation (14). The detection of 583 autosomal strain bias genes was a similar number to other studies that employed RNA-seq to investigate eQTLs in mouse adipose tissue (66) and adult liver (28). The number of strain bias genes detected in our study was over 20-fold higher than the number of imprinted genes, illustrating the importance of reciprocal crosses to detect true imprinted expression, and identifying genes that may explain the differences in the phenotype between the CAST and FVB strains. However, only 29% of strain biased genes were validated by the H3K4me3 ChIP-seq analysis, in contrast to the high validation rate of imprinted genes mentioned above, and a 90% validation rate for biallelic genes. Around 46% of genes categorized as strain-biased in RNA-seq showed biallelic H3K4me3 marks. Therefore, it is possible that in some cases the strain-biased levels of these transcripts arose not from allele specific transcription, but rather from allele specific post-transcriptional processing, for example, alternative splicing or alternative UTR generation, or due to strain-biased effects on miRNA-binding and RNA stability (10–12). Besides defining a set of genes that can be used as controls for studies of imprinted and strain biased genes, the identification of biallelically expressed genes can also be of interest in itself. For example, in our study we identified 120 biallelic genes on the X-chromosome despite the bias in X-inactivation. Of these genes 48 were validated by ChIP-seq as showing biallelic expression, including five of nine known X-inactivation escaper genes in our annotation (67). If the allelic cutoff for RNA-seq and ChIP-seq was reduced to 0.6 then seven biallelic genes were detected including four known escapers, making the remaining three genes strong novel X-inactivation escaper candidates.

In summary, using Allelome.PRO we were able to define the entire allelic expression status of all RefSeq genes from RNA-seq data. Validation of this allelome by differential H3K4me3 enrichment detected from ChIP-seq data created a high confidence set for each category of allelic expression. We also demonstrated that a high confidence allelome could be generated from RNA-seq data alone by changing the user-set minreads parameter in Allelome.PRO, resulting in lowly expressed genes from all categories being classified as non-informative.

### Applications of the Allelome.PRO pipeline

Most imprinted genes show tissue-specific imprinted expression, the pattern of which has only been relatively comprehensively characterized for a small number (25,68). Allelome.PRO in conjunction with RNA-seq, and validation by H3K4me3 ChIP-seq, provides a robust and sensitive method to assay a wide range of tissues and developmental time points, thus providing a complete picture of tissue-specific imprinted expression. In addition to known imprinted genes, novel tissue-specific imprinted genes may be uncovered in tissues that have not been thoroughly examined for imprinted expression previously. Such an approach would also classify strain biased genes into those that are found in multiple tissues, and those that show tissue-specificity and are therefore candidates to explain strain difference phenotypes in a particular organ or tissue.

Expression quantitative trait loci (eQTL) are defined as genomic loci that regulate gene expression and can be identified by combining whole genome association studies (GWAS) with differential expression analysis (10). Differentially expressed genes can be identified by differential expression analysis between two genotypes, or by allelic expression analysis from RNA-seq data (28,66). Mapping of *cis*-regulatory regions that may explain differences in expression then requires several generations of breeding from inbred strains in order to generate haplotypes that can then be subject to linkage analysis (10). In this study we demonstrated that Allelome.PRO could detect differential enrichment of H3K4me3 over promoters, indicating that it could also be used to detect differential enrichment in the genome of other histone modifications or chromatin binding proteins from ChIP-seq data. Allelic enrichment of enhancer marks, such as H3K27ac or H3K4me1, could identify eQTLs or enhancers that may regulate nearby strain bias genes detected by RNA-seq. This approach has the advantage over conventional eQTL analysis in that analysis is focused on enhancers rather than on all genetic variation between strains. Additionally, analysis can be conducted on the F1 generation, avoiding the extra breeding required for linkage analysis.

In summary, Allelome.PRO is a novel user-friendly pipeline to investigate allele specific features in high-throughput data using any compatible annotation and SNP file. In this study we showed the use of Allelome.PRO on expression and histone mark data, but allele specific differences of other features like DNA methylation or transcription factor binding could be investigated as well. Furthermore this pipeline is not limited to just one organism but instead it could be used in reciprocal crosses of strains from any given organism as long as a database of SNPs is available to distinguish the two alleles. By integrating analysis of different genomic features, such as expression and histone modifications, Allelome.PRO could be used as part of a toolset to investigate allele specific gene regulation.

## ACCESSION NUMBERS

RNA-seq and ChIP-seq data are deposited in the Gene Expression Omnibus (GEO) with the accession number GSE69168. Analyzed data can be viewed on the UCSC genome browser at the following link: https://opendata.cemm.at/barlowlab/. The Allelome.PRO program can be downloaded from the following link: https://sourceforge.net/projects/allelomepro/.

## Supplementary Material

SUPPLEMENTARY DATA
