# Supplementary material for: Allelome.PRO, a pipeline to define allele-specific genomic features from high-throughput sequencing data
Source: Nucleic Acids Res. 2015 Jul 21;43(21):e146. doi: 10.1093/nar/gkv727 (PMC4666383; doi:10.1093/nar/gkv727)
Supplement: SUPPLEMENTARY DATA [file supp_43_21_e146__index.html]

Allelome.PRO, a pipeline to define allele-specific genomic features from high-throughput sequencing data — Allelome.PRO, a pipeline to define allele-specific genomic features from high-throughput sequencing data — Allelome.PRO, a pipeline to define allele-specific genomic features from high-throughput sequencing data — SUPPLEMENTARY DATA 

# Allelome.PRO, a pipeline to define allele-specific genomic features from high-throughput sequencing data

## SUPPLEMENTARY DATA

- SUPPLEMENTARY DATA
- SUPPLEMENTARY DATA
- SUPPLEMENTARY DATA
- SUPPLEMENTARY DATA
- SUPPLEMENTARY DATA
