# Supplementary material for: Allelome.PRO, a pipeline to define allele-specific genomic features from high-throughput sequencing data
Source: Nucleic Acids Res. 2015 Jul 21;43(21):e146. doi: 10.1093/nar/gkv727 (PMC4666383; doi:10.1093/nar/gkv727)
Supplement: SUPPLEMENTARY DATA [file supp_gkv727_nar-01599-met-n-2015-File010.pdf]

Andergassen D, Dotter CP et. al
supp. Fig. 1. Sequencing error saturation curve

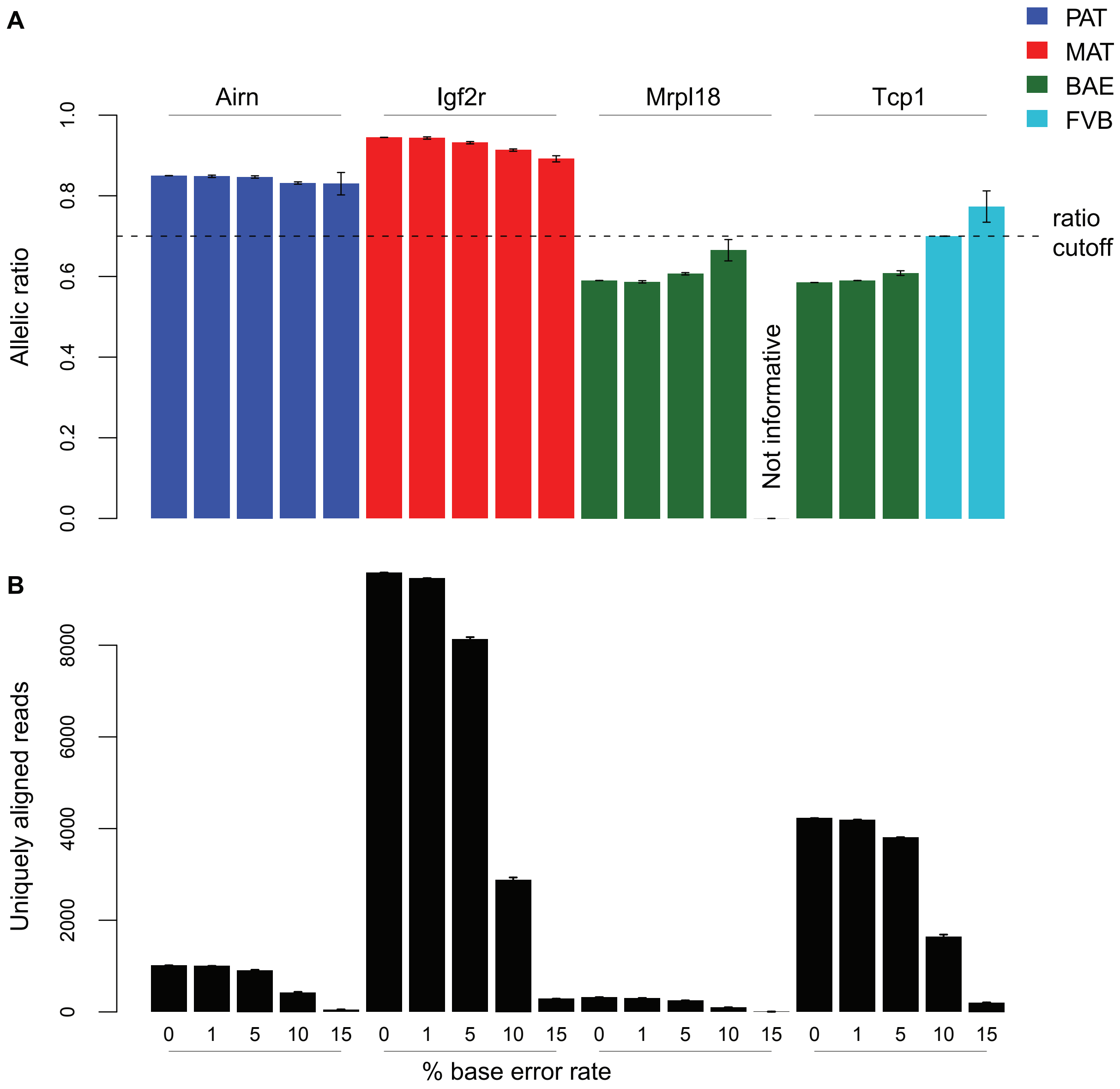

Figure S1. Allelic ratios generated by Allelome.PRO are robust over a wide range of sequencing error rates

(A) Allelic ratios of genes in the Igf2r region at different sequencing error rates. The imprinted ratio is plotted for *Airn* and *Igf2r*, and the strain biased ratio for *Mrpl18* and *Tcp1*. PAT paternal, MAT maternal, BAE biallelic, FVB strain bias.

(B) The number of uniquely aligned reads for each gene at different sequencing error rates.

Error bars show the standard deviation from generating random sequencing errors 3 times.
