# Supplementary material for: Allelome.PRO, a pipeline to define allele-specific genomic features from high-throughput sequencing data
Source: Nucleic Acids Res. 2015 Jul 21;43(21):e146. doi: 10.1093/nar/gkv727 (PMC4666383; doi:10.1093/nar/gkv727)
Supplement: SUPPLEMENTARY DATA [file supp_gkv727_nar-01599-met-n-2015-File011.pdf]

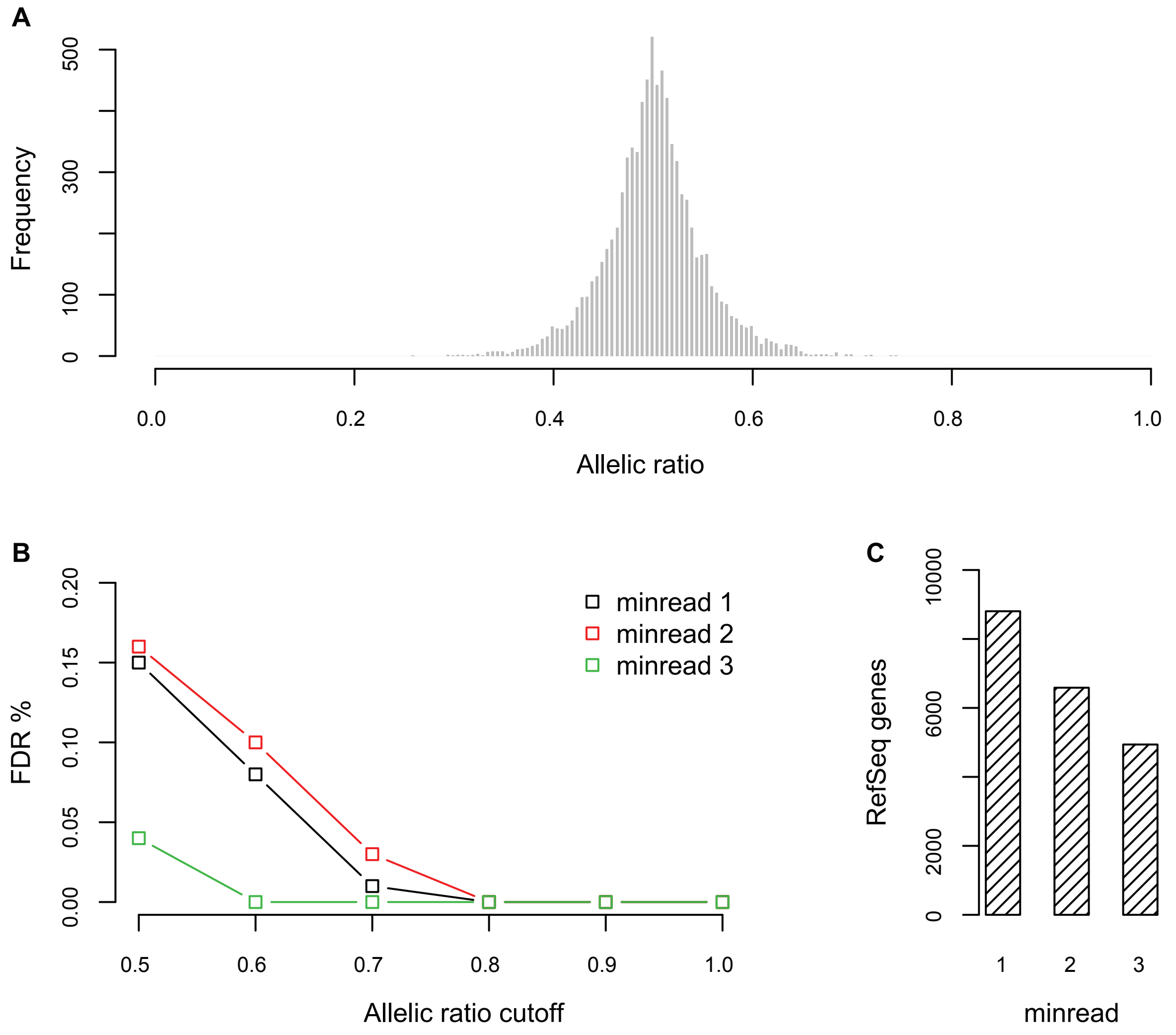

Figure S2. Determination of experimental error by in silico mixing of RNA-seq data from FVB/N and CAST/EiJ adult heart.

(A) Allelic ratio distribution of informative RefSeq genes generated by Allelome.PRO from a 50:50 mix of uniquely aligned reads from FVB and CAST.

(B) Determination of imprinted expression false discovery rate (FDR), defined as the percentage of informative genes called imprinted by Allelome.PRO at different allelic ratio cutoffs and minread parameter settings. The FDR is reduced when the minread parameter or the allelic ratio cutoff is increased.

(C) Increasing the minread parameter decreases the number of informative genes. The number of informative RefSeq genes at different minread settings is shown for an allelic ratio cutoff of 0.7.
