# Supplementary material for: Allelome.PRO, a pipeline to define allele-specific genomic features from high-throughput sequencing data
Source: Nucleic Acids Res. 2015 Jul 21;43(21):e146. doi: 10.1093/nar/gkv727 (PMC4666383; doi:10.1093/nar/gkv727)
Supplement: SUPPLEMENTARY DATA [file supp_gkv727_nar-01599-met-n-2015-File012.pdf]

Allelome.PRO

Defining allele-specific expression in high throughput sequencing data

MANUAL

Daniel Andergassen & Christoph Dotter

Contents

Table of contents **i**

1 Introduction **1**

2 Installation **1**

2.1 Hardware requirements 1

2.2 Software dependencies 1

2.3 Allelome.PRO content 2

3 Usage **3**

3.1 Input file requirements 3

3.1.1 The annotation file 4

3.1.2 The aligned BAM files 4

3.1.3 The SNP file 5

3.2 The configuration file 5

3.3 Run 7

3.3.1 Strand-specific analysis as performed in Andergassen and Dotter et al . 7

3.4 Output 7

3.4.1 Result tables 8

3.4.2 Graphical output 9

3.4.3 Result bed files 10

Bibliography **12**

1 Introduction

Allelome.PRO was developed in the group of Denise Barlow at the CeMM Research Center of Molecular Medicine of the Austrian Academy of Sciences (Vienna, Austria) as a fully automated user-friendly bioinformatics pipeline which uses high throughput sequencing data of four tissue samples from reciprocal crosses from genetically distinct mouse strains to detect allele-specific features. These features include allele-specific expression and allele-specific histone marks as demonstrated in the original publication:

Andergassen D, Dotter CP, Kulinski TM, Guenzl PM, Barlow DP, Pauler FM and Hudson QJ. Allelome.PRO, a pipeline to define allele-specific genomic features from high-throughput sequencing data. (Manuscript submitted 2015) [1]

When using this tool for a publication please cite the original publication.

2 Installation

2.1 Hardware requirements

During the runs of Allelome.PRO for the original publication we ran Allelome.PRO for data of one strand from strand specific RNA sequencing data using the RefSeq annotation. This translated into on average 41.8 million reads per sample for an annotation of around 15,000 genes per run, with a SNP file containing around 20 million SNPs. For these runs we observed that Allelome.PRO allocated a maximum of 3 GB of memory. The pipeline was designed to run inside a computer cluster environment with sufficient memory but runs also on a computer with less resources (tested on an iMac 5.1, 3Gb RAM, Dual-core 2.16Ghz processor).

2.2 Software dependencies

The pipeline was designed for Linux-like operating systems and was tested on Linux and Mac OS X.

Software required by the core pipeline

The pipeline requires the following programs/toolsets:

- bedtools ($\geq$ version 2.20.1) [2]
- SAMtools ($\geq$ version 0.1.19) [3]
- R ($\geq$ version 3.0.2) [4] + plyr package (will be automatically installed if internet access is possible)
- Perl ($\geq$ version 5.20.0)

All required software has to be located within the paths provided by the `PATH` environment variable. For instructions on how to set the PATH variable for your system please refer to one of the following pages:

- Instructions for setting the PATH variable in Linux/UNIX based systems
- Instructions for setting the PATH variable in Mac OS X.

Suggested additional software

For the alignment of RNA sequencing data as well as ChIP sequencing data we suggest the use of the STAR aligner (version $\geq$ 2.3.1) [5]. This is based on a comparison of three different aligners as described in the original publication.

2.3 Allelome.PRO content

The program archive is available at https://sourceforge.net/projects/allelomepro/. The archive contains the main pipeline shell script `allelome_pro.sh` as well as a folder `scripts` which contains scripts used by the main pipeline. In addition to that a helper script to create the SNP bed file (see 3.1.3) is included. A summary of all deployed files is given in table 1.

Script	Description
<base>/	
allelome_pro.sh	The main script that is **called by the user**.
<base>/scripts/	
bamtrim.sh<br>bamtrim.pl	Trim reads covering multiple SNPs so they just cover one.<br>This is done to prevent multiple counting of reads.<br>(for details please refer to the publication).
pileup_filter.pl	Handles spliced reads/indels in the pileup file.
read_count.pl	Sums up number of reads for each variant at SNP positions.
score.R	Statistical scoring and categorisation of the candidates.
bed_creator.sh	Creates color-coded output bed files.
<base>/helperscripts/	
createSNPbedfile.sh	Prepares the SNP input file (see chapter 3.1.3).<br>usage: `createSNPbedfile.sh <vcf_file> <snp_file>`
separate_BAM_strand.pl	Divides reads from an aligned BAM file into two files<br>usage: `separate_BAM_strand.pl`<br>containing reads from the forward and reverse strand,<br>respectively (see chapter 3.3.1).

Table 1: Allelome.PRO content. This table lists the scripts that are part of the Allelome.PRO core pipeline and shortly describes their purpose. All scripts besides the main script are located in the `scripts` folder.

3 Usage

The usage of Allelome.PRO requires three steps:

1. Prepare the required input files.
2. Set up the configuration file for the needs of your analysis
3. Run the pipeline

3.1 Input file requirements

The pipeline requires three types of input files: An annotation file, four BAM files containing the aligned sequencing data and a file containing information about the SNPs between the crosses used. The exact format requirements along with examples for each will be described in the next three sections.

3.1.1 The annotation file

The annotation file is in BED6 format (see UCSC format description for more details), meaning it has six columns containing the information listed in table 2. In the simplest case it contains one line per candidate that should be categorised. It is also possible to include multiple lines per candidate (e.g. multiple PCR products for one gene, multiple isoforms of the same gene in an annotation such as RefSeq) which will be combined to one during the analysis. This is made possible by the fact that the pipeline groups lines in the annotation if they have the same name and are located on the same chromosome and strand, summing up SNPs covered by at least one of the grouped entries. Users should keep this grouping feature in mind when they curate their annotation to avoid errors. If, for example, the user wants to score different isoforms independently the names in the annotation will need to be different from each other (e.g. start with consecutive numbers).

Column	Information
1	Chromosome (written as e.g. chr1, chrX)
2	Start Position
3	End Position
4	Name (e.g. gene name)
5	Score (not used here)
6	Strand (e.g. +,- or . for not defined)

Table 2: The BED6 format. This format is used for both the annotation file and the SNP file.

To give some examples, here are the annotations used by the original publication:

- The "RefSeq Genes" annotation [6] (obtained via the UCSC table browser [7]).
- Sliding window annotations over the whole genome.
- A custom annotation of RefSeq Gene promoter regions.

3.1.2 The aligned BAM files

The pipeline requires four aligned BAM files derived from samples of two reciprocal crosses with two samples being from one cross, while the other two are from the other cross. This was tested for samples from RNA sequencing and ChIP sequencing experiments on an Illumina® sequencing system. The BAM files are ideally sorted by

leftmost coordinates (as done by samtools sort), but we also implemented an option to sort the BAM files before processing (see section 3.2).

3.1.3 The SNP file

The SNP file is also in BED6 format (see table 2) with the additional requirement that the name consists of only two letters indicating the two SNP variants present in the two strains of the crosses. The order of these two letters is important. The first letter indicates the variant in strain 1, while the second one indicates the variant in strain 2. **The way the SNP file is created therefore defines which strain will be "strain 1" and which one will be "strain 2" during the course of the analysis.** This should be kept in mind as some parameters in the configuration file need to be set according to this definition. SNP positions have to be based on the same reference genome that the BAM files were aligned to (e.g. mm10). One source for SNP data is the FTP site of the Sanger institute. The downloaded compressed VCF file (e.g. `mgp.v3.snps.rsIDdbSNPv137.vcf.gz` as used in the publication) can then be extracted and further processed using the included helper script `createSNPbedfile.sh`. The script takes two parameters, the first one being the VCF file, the second one being the desired SNP bed file name. Once started it lists all the strains for which the VCF file contains information and lets the user select the strains he wants to use. Afterwards it extracts the variant information for each SNP with different variants in the two crosses. Only high confidence SNPs homozygous in both strains are considered.

Once all input files are prepared, proceed with setting up the configuration file.

3.2 The configuration file

The configuration file is written in the parameter=value format (note: no spaces allowed). The available parameters, along with a short description are listed in table 3. A template configuration file containing documentation is included in the distribution.

General Parameters	
`pipe_location`	Complete path to the folder where the main pipeline script is located.
`ratio`	Minimum allelic ratio above which allele-specific expression is called biologically relevant.<br>Default value: 0.7 - represents a 70:30 ratio between the two alleles.
`fdr_param`	The false-discovery rate set as how many candidates were called allele-specific in the mock analysis compared to the results.<br>Default value: 1 - represents 1%, meaning that for each 100 genes categorised as allele-specific in the results, one call is allowed in the mock comparison
`minreads`	Minimum number of reads that must cover a SNP position for the SNP to be included in the analysis.<br>Default value: 1 - include all covered SNPs.

Experiment-specific Parameters	
`outputdir`	Path where the job directory containing all output files will be created. The job directory name will contain the date, sample file names, annotation file name, and the FDR and minreads parameter.
`annotation`	Annotation file containing the candidates to analyse (see 3.1.1).
`main_title`	Title of the analysis, used for plot captions and file names.<br>Note: No spaces or special characters are allowed here.
`y_axis`	Y axis for the result plot, describes the type of candidates.<br>Examples: RefSeq Genes, windows.
`sorted`	Specify whether the BAM files are sorted or not.<br>Default value: 1 - the files are sorted; 0 - unsorted.

Cross-specific Parameters	
`snp_file`	The SNP file used in this analysis (see 3.1.3).
`strains`	Labels for the two strains, separated by a semicolon (;).<br>Example: CAST;FVB
`for_c1`<br>`for_c2`<br>`rev_c1`<br>`rev_c2`	The four BAM files containing the aligned sequencing data. for_c1 and for_c2 are the samples of the forward cross, meaning the cross where the mother is of strain 1 and the father is of strain 2. rev_c1 and rev_c2 are the samples of the reverse cross in the opposite direction. Strains are defined via SNP file (see 3.1.3).

Table 3: Parameters in the configuration file.

3.3 Run

Syntax for starting the pipeline:
`<pipeline-dir>/allelome_pro.sh -c <path-to-configfile>`

3.3.1 Strand-specific analysis as performed in Andergassen and Dotter et al

It is well accepted that genes show complex spatial organization resulting in transcription from the same genomic region albeit from different DNA strands. These overlapping transcription units can show profound differences in their allelic expression pattern. To resolve this complexity RNA-Seq methodologies have been developed that retain the information of the strand that a particular RNA was transcribed from. To keep the core pipeline of Allelome.PRO as simple as possible we have not implemented an automatic "strand specific" analysis yet. We describe a workflow in the original publication that is based on the separate analysis of RNA-Seq reads originating from the forward and from the reverse strand and provide the necessary script to follow this workflow in this package. This script, `separate_BAM_strand.pl` can be found in the helperscripts folder of this package. The syntax for the script is as follows: `separate_BAM_strand.pl <bam_file> <strand_rule> <output_folder>` and it creates two files named `<bam_file>.fwd.bam` and `<bam_file>.rev.bam`. The strand rule indicates how the reads should be divided. For more documentation on the choice of strand rule please refer to the documentation in the header of the script. The pipeline can then be started for each strand separately. In addition to the required separation of reads it is also advisable to split the used annotation into forward and reverse strand as well and using the matching annotation for each of the two pipeline runs. Afterwards the results can be combined by concatenating the respective result tables. An option to generate combined graphical output is not implemented yet.

3.4 Output

The result directory contains the files and folders listed in table 4

Name	Description
<main>/	
<main_title>_IG.txt	Contains all loci categorised as imprinted after both FDR and ratio filtering.
<main_title>_SG.txt	Contains all loci categorised as strain biased after both FDR and ratio filtering.
<main_title>_locus_full.txt	Information about the categorisation of all loci in the annotation.
<main_title>_SNP_full.txt	Information about the categorisation of all SNPs in the annotation.
<main_title>.pdf	Graphical output of the allelome data.
info.txt	Additional information about the run
BED_files/	
<main_title>_locus.bed	BED6-file containing all loci, color-coded according to their categorisation
<main_title>_SNP.bed	BED6-file containing all SNPs, color-coded according to their categorisation
debug	Folder containing all files created during the run.

Table 4: Result files and folders. All resulting files and folders created by the pipeline. <main> represents the main output folder created by the pipeline, while <main-title> represents the title specified in the configuration file.

3.4.1 Result tables

The four result tables can be separated into two groups. First, `<main_title>_IG.txt` and `<main-title>_SG.txt` contain only the respective subset of the annotated loci categorised as showing imprinted and strain biased expression, respectively. The columns are listed in table 5. Colums 8 and 9 are different between the two files, with `<main_title>_IG.txt` containing I_score and I_ratio while `<main_title>_SG.txt` contains S_score and S_ratio.

Columns 1-6 of the files `<main_title>_locus_full.txt` and `_SNP_full.txt` are the same as listed in table 5. The seventh column, total_reads_min, shows the minimum number of reads covering SNPs in this locus across the four replicates. Column 8 is the same RPSM_min column as before, columns 9 and 10 contain the imprinting score and ratio, columns 11 and 12 contain the strain score and ratio and column 13 contains the tag.

Nr.	Column	Description
1	**chr**	
2	**start**	
3	**end**	Locus information from the annotation file.
4	**name**	
5	**strand**	
6	**cov_SNP_min**	Minimum number of SNPs covered in a single biological replicate.
7	**RPSM_min**	Minimum RPSM (**R**eads **P**er **S**NP per **M**illion total SNP covering reads) value across the four replicates. This gives an estimate of the expression level of the locus.
8	**I_score** or **S_score**	The "imprinting score", calculated as allelic score between maternal and paternal allele or the "strain score", calculated as allelic score between the alleles of strains 1 and 2. These scores are equal to the minimum score across the four replicates.
9	**I_ratio** or **S_ratio**	Average maternal:total ratio across the four replicates. Average strain1:total ratio across the four replicates.
10	**tag**	Result of the categorisation.

Table 5: Result table columns. Listed are the columns of the files `<main-title>_IG.txt` and `<main-title>_SG.txt`, together with a short description of the information contained in them.

3.4.2 Graphical output

The pdf file produced by the pipeline, `<main_title>.pdf`, contains five different plots.

1. A barplot displaying the overall results of the categorisation.
2. A stacked barplot visualising the allelic ratios of the candidates defined as showing imprinted expression.
3. A graph illustrating the calculation of the score cutoff for the loci based on the false discovery rate (for more information please refer to the paper).
4. The same graph for the SNPs.
5. A graph showing the distribution of allelic ratios among the candidates with a score significant enough to pass the FDR cutoff. This graph can be used to determine whether the set ratio cutoff was a good choice and should aid in setting an allelic ratio cutoff. The upper graph shows the imprinted genes, while the bottom one shows strain biased genes.

Since X inactivation in females represents a special case of monoallelic expression, candidates from chromosome X are handled in a special way in these plots.

Chromosome X candidates in plot 1:
Candidates on chromosome X which were categorised as either imprinted or strain biased are displayed as lighter bars stacked atop the autosomal candidates. The numbers above the stacked bars represent the total numbers of imprinted and strain biased candidates from all chromosomes.

Chromosome X candidates in plots 2-4:
In these plots candidates from chromosome X are omitted. This is because imprinted genes on chromosome X are most likely the result of parental specific X inactivation (e.g. in extra-embryonic mouse tissues) and not themselves regulated in an imprinted fashion. This is also the reason why imprinted and strain biased candidates from chromosome X are not included in the calculations for the FDR cutoff.

Chromosome X candidates in plot 5:
Here the chromosome X candidates are not displayed in the plot for the imprinted genes (top) but indicated in the plot for the strain biased genes (bottom). This behaviour was chosen because the amount of imprinted chromosome X genes in a situation of parental specific X inactivation is very high compared to the number of autosomal imprinted candidates. This would distort the ratio distribution.

3.4.3 Result bed files

The two BED9 files `<main_title>_locus.bed` and `<main_title>_SNP.bed` include the data of the full result tables `<main_title>_locus_full.txt` and `_SNP_full.txt`, respectively. These files were created to include the categorisation of the candidates via a color code in the color column of the bed file. Furthermore information about relevant ratios and read counts are encoded in the name column. This is done by appending one or more of the following suffixes to the name of the locus/SNP:

- `_i<ratio>`: `<ratio>` gives the ratio of reads supporting the maternal variant over total reads (shown for imprinted and biallelic loci/SNPs).
- `_s<ratio>`: `<ratio>` gives the strain1 reads/total reads ratio (shown for strain biased and biallelic loci/SNPs).
- `_r<number>`: `<number>` gives the minimum number of SNP covering reads across the four replicates (shown for all loci with SNPs/all SNPs).

- `<var1>|<rc_v1_fwd>|<rc_v1_rev>||<var2>|<rc_v2_fwd>|<rc_v2_rev>`:
  Variant info (SNP file only) providing information about the two SNP variants (`<var1>`/`<var2>`) and the read counts in the forward (`<rc_v1_fwd>`, `<rc_v2_fwd>`) and the reverse cross (`<rc_v1_rev>`, `<rc_v2_rev>`) for variant 1 and 2, respectively. Read counts were summed up across the two replicates for each cross to enhance readability.

These BED files can then be used to visualise the results using a genome browser of choice (e.g. the UCSC genome browser) by uploading them as custom tracks.

Bibliography

[1] Andergassen D, Dotter CP, Kulinski TM, Guenzl PM, Barlow DP, Pauler FM, et al. Allelome.PRO, a pipeline to define allele-specific genomic features from high-throughput sequencing data. submitted. 2015;.

[2] Quinlan AR, Hall IM. BEDTools: a flexible suite of utilities for comparing genomic features. Bioinformatics. 2010 Mar;26(6):841–842.

[3] Li H, Handsaker B, Wysoker A, Fennell T, Ruan J, Homer N, et al. The Sequence Alignment/Map format and SAMtools. Bioinformatics. 2009 Aug;25(16):2078–2079.

[4] R Core Team. R: A Language and Environment for Statistical Computing. Vienna, Austria; 2013.

[5] Dobin A, Davis CA, Schlesinger F, Drenkow J, Zaleski C, Jha S, et al. STAR: ultrafast universal RNA-seq aligner. Bioinformatics. 2013 Jan;29(1):15–21.

[6] Pruitt KD, Brown GR, Hiatt SM, Thibaud-Nissen F, Astashyn A, Ermolaeva O, et al. RefSeq: an update on mammalian reference sequences. Nucleic Acids Res. 2014 Jan;42(Database issue):D756–D763.

[7] Karolchik D, Hinrichs AS, Furey TS, Roskin KM, Sugnet CW, Haussler D, et al. The UCSC Table Browser data retrieval tool. Nucleic Acids Res. 2004 Jan;32(Database issue):D493–D496.
